# PRMT5-mediated methylation of YBX1 regulates NF-κB activity in colorectal cancer

**DOI:** 10.1038/s41598-020-72942-3

**Published:** 2020-09-28

**Authors:** Antja-Voy Hartley, Benlian Wang, Rasika Mundade, Guanglong Jiang, Mengyao Sun, Han Wei, Steven Sun, Yunlong Liu, Tao Lu

**Affiliations:** 1 Department of Pharmacology and Toxicology, Indiana University School of Medicine, 635 Barnhill Drive, Indianapolis, IN 46202 USA; 2 Case Western Reserve University, Cleveland, OH USA; 3 Department of Biochemistry and Molecular Biology, Indiana University School of Medicine, Indianapolis, IN USA; 4 Department of Medical and Molecular Genetics, Indiana University School of Medicine, Indianapolis, IN USA

**Keywords:** Biological techniques, Cancer, Cell biology, Molecular biology

## Abstract

The multifunctional protein Y-box binding protein 1 (YBX1), is a critical regulator of transcription and translation, and is widely recognized as an oncogenic driver in several solid tumors, including colorectal cancer (CRC). However, very little is known about the upstream or downstream factors that underlie YBX1′s regulation and involvement in CRC. Previously, we demonstrated that YBX1 overexpression correlated with potent activation of nuclear factor κB (NF-κB), a well-known transcription factor believed to be crucial in CRC progression. Here, we report a novel interaction between NF-κB, YBX1 and protein arginine methyltransferase 5 (PRMT5). Our findings reveal for the first time that PRMT5 catalyzes methylation of YBX1 at arginine 205 (YBX1-R205me2), an event that is critical for YBX1-mediated NF-κB activation and its downstream target gene expression. Importantly, when WT-YBX1 is overexpressed, this methylation exists under basal (unstimulated) conditions and is further augmented upon interleukin-1β (IL-1β) stimulation. Mechanistically, co-immunoprecipitation studies reveal that the R205 to alanine (A) mutant of YBX1 (YBX1-R205A) interacted less well with the p65 subunit of NF-κB and attenuated the DNA binding ability of p65. Importantly, overexpression of YBX1-R205A significantly reduced cell growth, migration and anchorage-independent growth of CRC cells. Collectively, our findings shed important light on the regulation of a novel PRMT5/YBX1/NF-κB axis through PRMT5-mediated YBX1-R205 methylation. Given the fact that PRMT5, YBX1 and NF-κB are all among top crucial factors in cancer progression, pharmacological disruption of this pivotal axis could serve as the basis for new therapeutics for CRC and other PRMT5/YBX1/NF-κB-associated cancers.

## Introduction

As part of the family of multifunctional DNA/RNA binding proteins, YBX1 plays critical roles in a wide range of cellular functions including transcriptional regulation^1^, DNA repair^2^, splicing^3^, mRNA translation and stability^4^ as well as cellular stress responses^5^. Due to its involvement in these important functions, it is unsurprising that dysregulation of YBX1 is frequently linked to diseases such as cancer. In fact, its overexpression has been strongly associated with certain “hallmark” features such as malignant growth, therapeutic resistance, invasion, metastasis, aberrant cell proliferation and overall poor patient prognosis across several tumor types^6^. These include but are not limited to cancers of the breast^7^, bladder^8^, prostate^9^ and colon^10, 11^. The diverse biological functions of YBX1 and its involvement in cancer appear to arise not only from its broad nucleic acid binding properties, but may be dependent on its cooperativity with other signaling pathways and molecules responsible for cancer development and/or progression. For instance, recent reports indicate that the expression of YBX1 and a Ras GTPase-activating protein-binding protein 1 (G3BP1), a protein involved in a variety of growth-related signaling pathways, such as p53 and Ras signaling, were highly correlated in sarcomas^12^. Moreover, YBX1 was shown to directly interact with G3BP1, promoting enhanced migration and invasion of renal cell carcinoma (RCC) cells via a Secreted Phosphoprotein 1 (SPP1)/NF-κB signaling axis^12^. Intriguingly, our group previously showed that YBX1 overexpression could profoundly activate NF-κB signaling, and may thus serve as a novel therapeutic target for colorectal cancer (CRC) patients in which aberrant NF-κB activity is a major driver of malignancy^10, 11^.

Over the past decade, constitutive activation of NF-κB has been increasingly recognized as a major player in tumorigenesis, cancer progression and the development of chemoresistance in several cancers^13^. NF-κB proteins represent a family of inducible transcription factors characterized by their ability to orchestrate the expression of a large array of genes involved in essential cellular processes such as immune and inflammatory responses, differentiation, survival and proliferation^14^. The family consists of five proteins [RelA (p65), RelB, c-Rel, p50/p105 and p52/p100] that are structurally related based on the presence of an N-terminal domain essential for dimerization and binding to cognate DNA elements known as the Rel homology domain (RHD)^15^. In mammals, the five NF-κB members typically exist as heterodimers that are retained in the cytoplasm by their interaction with inhibitor of NF-κB (IκB) proteins^15^. In the context of canonical NF-κB signaling, p65/p50 represents the prototypical heterodimeric subunits, and upon stimulation of the cell with certain extracellular signals such as stress, cytokines [eg. Interleukin-1 beta (IL-1β)] and Lipopolysaccharides (LPS), IκB becomes proteasomally degraded and p65/p50 can then rapidly translocate to the nucleus to activate target gene transcription^16^.

Several reports indicate that NF-κB is dynamically regulated by a number of upstream factors, many of which help to promote its hyperactivity and tumor-promoting transcriptional functions^13, 17–19^. In addition to YBX1, we previously showed that the p65 subunit of NF-κB is also positively regulated by another important driver of tumor malignancy, protein arginine methyltransferase 5 (PRMT5)^10, 11^. We found that signal-dependent dimethylation of R30 of the p65 subunit NF-κB by PRMT5 could profoundly affect the transient binding of NF-κB to κB response elements to activate gene transcription^17^. PRMT5, a member of the protein arginine methyltransferase (PRMT) superfamily, catalyzes the symmetric dimethylation of arginine residues on both histones and non-histone proteins^20^. In general, PRMTs are classified based on the type of methylation marks they catalyze. Type I PRMTs (PRMT 1, 2, 3, 4, 6, 8) predominantly catalyze asymmetric dimethylation of arginine residues, whereas type II enzymes (PRMT5, 9) mediate symmetrical dimethylation. Type III PRMTs (PRMT7) however preferentially catalyze only monomethylation marks^21^. PRMT5 is considered the major type II PRMT and functions by interacting with several cytoplasmic and nuclear molecules ranging from transcription factors (eg. p65, p53)^21^, splicing (eg. Sm proteins)^22^ and elongation factors (eg. Transcription elongation factor SPT50)^23^, among others. Importantly, through these interactions and its frequent overexpression in several tumor types including CRC, PRMT5 dysregulation has been linked to tumor cell proliferation, migration, epithelial-to-mesenchymal transition (EMT), invasion and metastasis^21^. It is therefore unsurprising that an increasing number of studies, including ours, have suggested that PRMT5 represents a novel, promising therapeutic anti-cancer target^24^. Specifically, knockdown or small-molecule-mediated PRMT5 inhibition significantly limits the growth of cancer cells to improve the overall prognosis of cancer patients^25^.

In the present study, we sought to further elucidate the complex molecular underpinnings of a previously unreported interaction between PRMT5 and YBX1 and how it relates to NF-κB, with the goal of uncovering novel therapeutic avenues for CRC. Previously, our group and others have identified phosphorylation modifications within the cold-shock domain (CSD) of YBX1 as essential contributors to increasing the complexity of its interactions and functional diversity in both normal and malignant cell types. For instance, Evdokimova et al.^26^ was the first to demonstrate that Akt-mediated phosphorylation of YB-1(YBX1) at S102 precluded binding of YBX1 to mRNA thereby relieving translational repression of oncogenes such as Insulin-like growth factor 1 (IGF-1), Vascular endothelial growth factor (VEGF) and Fos Proto-Oncogene (FOS) in breast cancer cells. More recently, we showed that IL-1β, but not IGF-1, induced the phosphorylation of YBX1 at S165 and S176, promoting its cytoplasmic retention^10, 11^. We now report for the first time that YBX1 is symmetrically dimethylated by PRMT5 at the highly conserved arginine 205 (R205) residue, a modification critical to the activation of NF-κB by YBX1. Overexpression of an R205A mutant downregulated expression of a subset of NF-κB target genes indirectly through attenuation of the DNA binding ability of p65 and YBX1-p65 interaction under both basal (unstimulated) and IL-1β-stimulated conditions. Moreover, the migration, anchorage-independent growth and cell growth of CRC cells were significantly compromised by overexpression of the R205A mutant compared to the wild type YBX1 (WT-YBX1). Our study thus reveals methylation as a novel post-translational modification (PTM) on YBX1, and a previously undiscovered functional cooperativity between YBX1 and PRMT5 that converges on NF-κB signaling. Together, these findings reveal a new mechanism underlying the finely-tuned regulation of specified gene networks involving the activation of a PRMT5/YBX1/NF-κB signaling axis.

## Results

### YBX1 is symmetrically dimethylated at R205 in response to IL-1β stimulation

**Figure 1:**
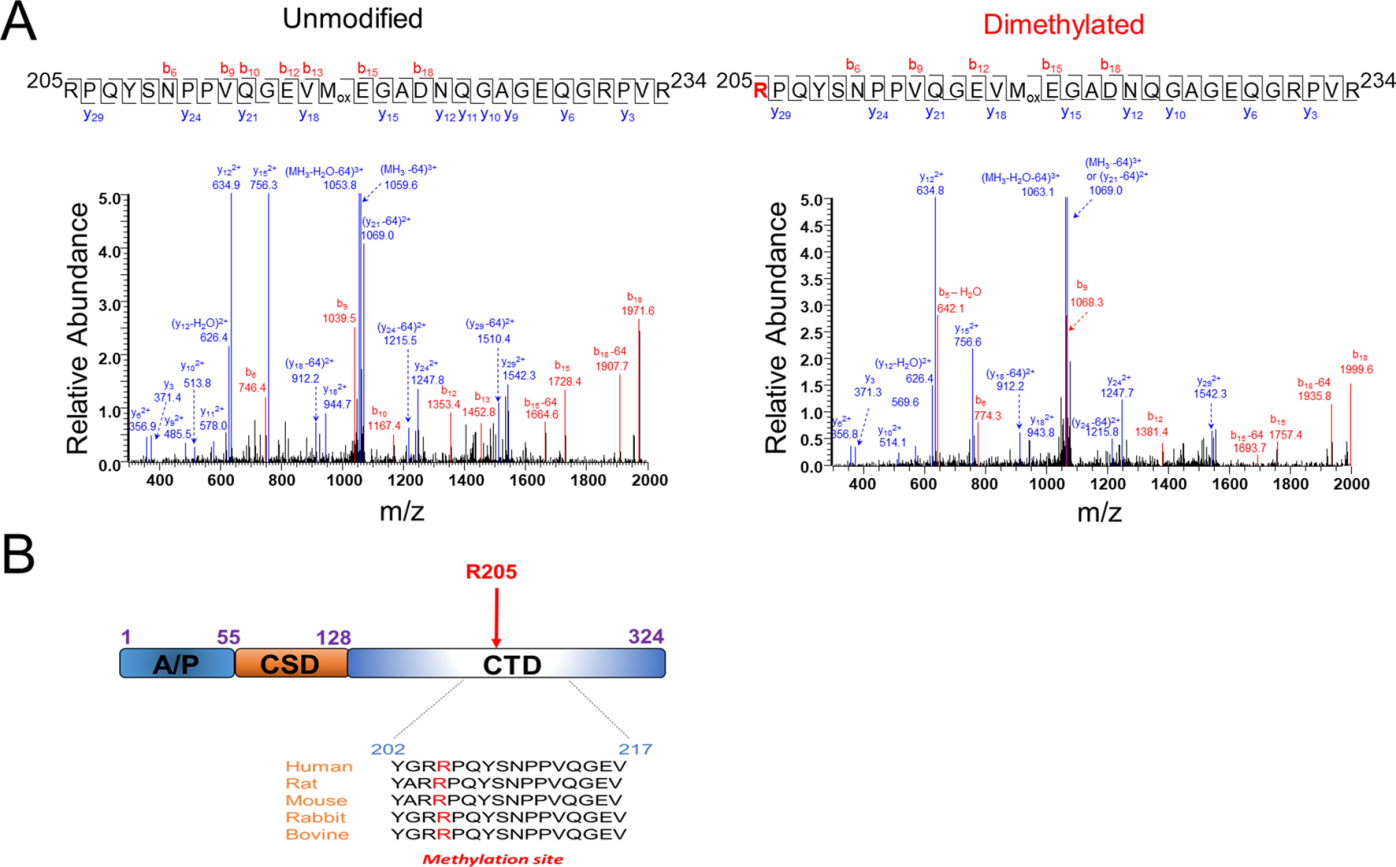
Identification of R205 on YBX1 as methylation site (**A**) *Left panel*, Tandem mass spectrum of YBX1 peptide, RPQYSNPPVQGEVM_ox_EGADNQGAGEQGRPVR (205–234) with a precursor ion of 1080.5170 (3 +) and R205 unmodified (left panel, mass error of 4 ppm) as well as a precursor ion of 1089.8540 (3 +) and R205 dimethylation (right panel, mass error of − 3 ppm). Mass loss of 64 Da indicates the neutral loss of methionine sulfoxide (CH3SOH) from oxidized methionine (M_ox_). (**B**) Domain organization of YBX1 shows Ala/Pro-rich N-terminal (A/P) domain (1–55); cold shock domain (CSD, 56–128); disordered C-terminal domain (CTD) harboring the conserved Arg 205 (R205) dimethylation residue (129–324).

Although YBX1 has been widely implicated in promoting cancerous phenotypes, the factors affecting its functions within these contexts remain poorly understood. In previous studies, our group and others have shown that YBX1 can be dynamically regulated by PTMs in cancers of the breast and colon^10, 11, 26^. To date, these reports have only demonstrated phosphorylation as playing a regulatory role in the translational functions and cellular localization of YBX1 in response to certain stimuli such as IGF-1 and more recently by us, IL-1β. However, whether other PTMs are involved in modulating additional aspects of its function is still unknown. To determine whether any additional PTMs exist on YBX1, we first purified Flag-tagged YBX1 using Flag-M2 beads from unstimulated and IL-1β-treated HEK293 and subjected the band corresponding to Flag-YBX1 to further mass spectrometry analyses. As shown in Fig. 1A (right panel), we found that a peptide with the sequence RPQYSNPPVQGEVM_ox_EGADNQGAGEQGRPVR (205–234) contained a dimethylation with a mass shift of 28 Da by the precursor ions of 1089.8540 (3 +) in contrast to 1080.5170 (3 +) of the unmodified peptide. There are three Rs in the peptide namely R205, R231, and R234. Further analysis of the fragmented ions indicates that the dimethylation is located at the first arginine residue R205, since the series of observed y ions from y3 to y29 are unaltered in comparison with the unmodified peptides. Furthermore, beside the y series, the mass difference of the b series also indicates the modification as occurring on the first R of the peptide. Alignment of YBX1 sequences from different mammalian species showed that this R205 site is located within the disordered C-terminal domain and is well conserved (Fig. 1B). Importantly, this R205 dimethylation was observed in both IL-1β treated and untreated samples with Flag-tagged WT-YBX1 overexpression.

### PRMT5 interacts with and catalyzes symmetric dimethylation of YBX1

**Figure 2:**
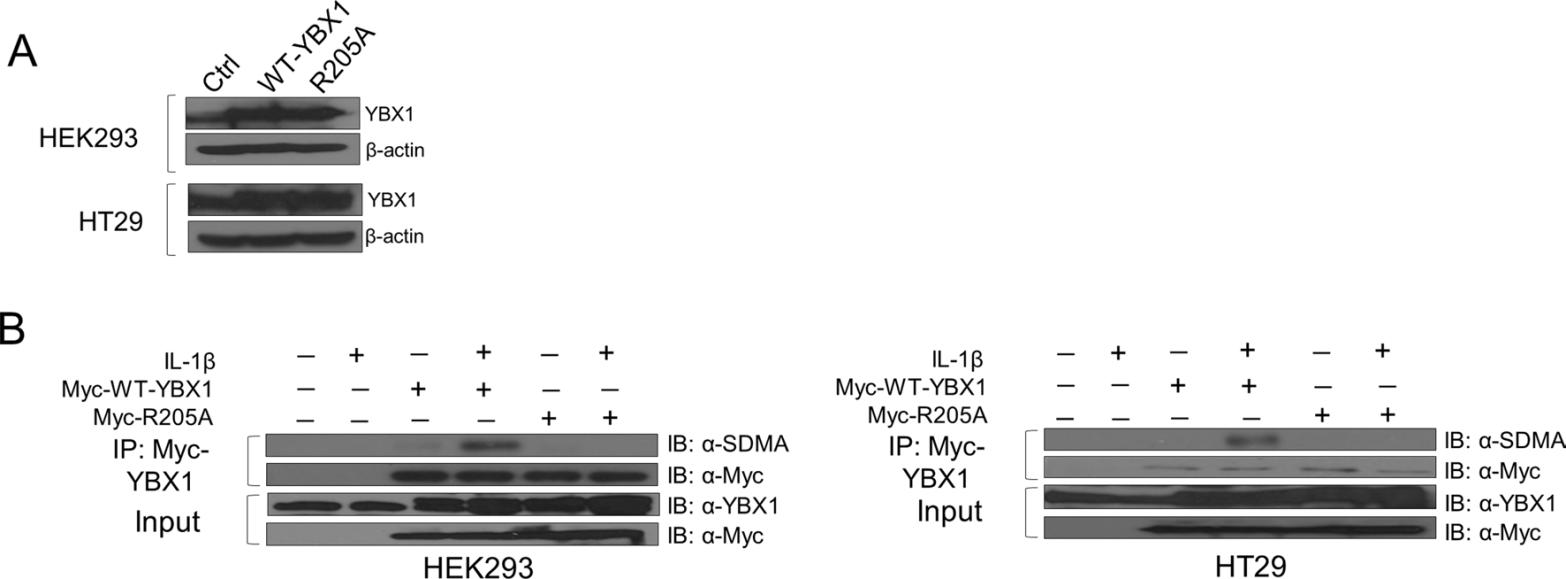
YBX1 is symmetrically dimethylated on R205 in response to IL-1β. (**A**) Western analysis of comparable expression of WT-YBX1 and R205A constructs stably overexpressed in HEK293 and HT29 cells. Anti-YBX1 and anti-β-actin images for each cell line were obtained from different immunoblots and developed on film at different exposures. (**B**) Detection of symmetric dimethylarginine (SDMA) on immunoprecipitated Myc-WT-YBX1 or Myc-R205A proteins stably expressed in HEK293 (*left*) and HT29 cells (*right*). Anti-Myc IP images were obtained from the same immunoblots as Anti-SDMA images that were stripped and reprobed for total Myc and developed at different exposures. Input images were developed from separate blots at different exposures.

**Figure 3:**
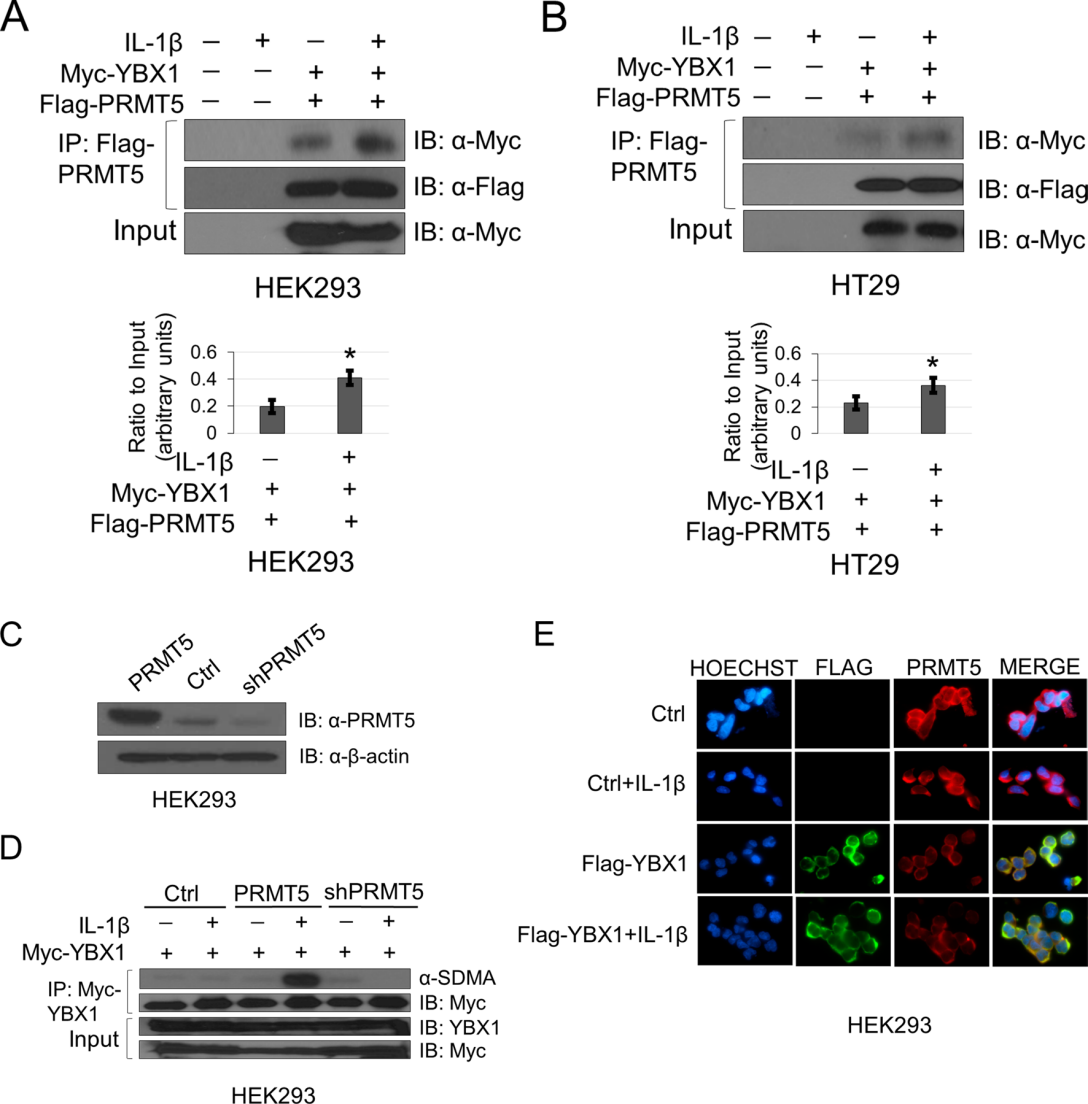
YBX1 interacts with, and is symmetrically dimethylated by PRMT5. (**A**) Representative co-immunoprecipitation (Co-IP) of Myc-YBX1 and Flag-PRMT5 shows that YBX1 and PRMT5 interact more strongly under IL-1β—stimulating conditions in HEK293 and (**B**) HT29 cells. Anti-Myc IP and Anti-Myc input images were obtained from the same blot with the same exposure. Anti-Flag IP images were obtained from the same blot that was stripped and reprobed with Anti-Flag antibody and developed at different exposures. First two lanes represent empty vector control stable lines compared to second two lanes which represent stable lines dually co-expressing Myc-YBX1 and Flag-PRMT5 constructs. Bottom panels, (**A**) and (**B**) show representative graphs of quantified western blot Myc co-IP images relative to input. (**C**) Western analysis to assess PRMT5 expression levels in cells expressing either Myc-YBX1 alone (Ctrl) or in combination with PRMT5 overexpression vector or shRNA-PRMT5 constructs. (**D**) Western analysis to assess dimethylation status of immunoprecipitated Myc-WT-YBX1 in cells with PRMT5 overexpression or its shRNA knockdown (shPRMT5), respectively. Anti-SDMA IP and Anti-Myc IP images were obtained from the same blot, stripped and reprobed for total Myc but developed at different exposures. Input images were obtained from different blots and developed at different exposures. (**E**) Immunofluorescence showing the localization of Flag-WT-YBX1 and PRMT5 with and without IL-1β treatment (10 ng/ul). Hoechst was used for nuclear staining. Images were taken at 63 × . *p < 0.05 Ratio of Myc-YBX1/Flag-PRMT5 Co-IP vs.YBX1/Flag-PRMT5 Co-IP + IL-1β stimulation relative to input. Densitometry analysis was performed using ImageJ software.

To assess the role of methylation of YBX1 at R205, we first generated an arginine-to-alanine mutant (R205A) and successfully stably overexpressed the R205A mutant at a level comparable with WT-YBX1 in HEK293 and HT29 cells (Fig. 2A). Since the mass spectrometry data did not directly inform us of the type of dimethylation (symmetric or asymmetric), we first identified the potential methyltransferase mediating R205 methylation. Using mass spectrometry protein identification studies, we revealed PRMT5 as a novel interacting partner of YBX1. We then further sought to confirm this YBX1-PRMT5 interaction by co-immunoprecipitation followed by western blot analyses. Additionally, since PRMT5 catalyzes symmetric dimethyl marks, we also determined whether YBX1 could in fact be symmetrically dimethylated. To facilitate this study, we generated the Myc-WT-YBX1 and Myc-R205A overexpressing stable cell lines. As shown in Fig. 2B, when Myc tagged-WT-YBX1 was overexpressed, it could be symmetrically dimethylated at basal level. However, upon further treatment with IL-1β, the symmetric dimethylation of the Myc-WT-YBX1 protein immunoprecipitated from HEK293 and HT29 cells was greatly enhanced whereas this was abolished on the Myc-R205A mutant protein, indicating that R205 constitutes a major symmetric dimethylation site on YBX1. Moreover, co-immunoprecipitation studies confirmed that YBX1 interacted more strongly with PRMT5 in response to IL-1β stimulation (Fig. 3A,B) using HEK293 and HT29 cells co-expressing Flag-PRMT5 and Myc-WT-YBX1 constructs. It is worth noting that even without IL-1β treatment, PRMT5 had a certain level of binding with YBX1 at basal level (Fig. 3A,B). Importantly, overexpression and shRNA-mediated knockdown of PRMT5 (Fig. 3C) correlated with enhanced and reduced symmetric dimethylation of YBX1, respectively (Fig. 3D). Furthermore, we observed that both proteins were shown to be localized predominantly in the cytoplasm by immunofluorescence experiments with no significantly observable change in subcellular distribution upon IL-1β treatment (Fig. 3E).

### R205 methylation is important for the activation of NF-κB by YBX1 and modulates p65 DNA binding ability

**Figure 4:**
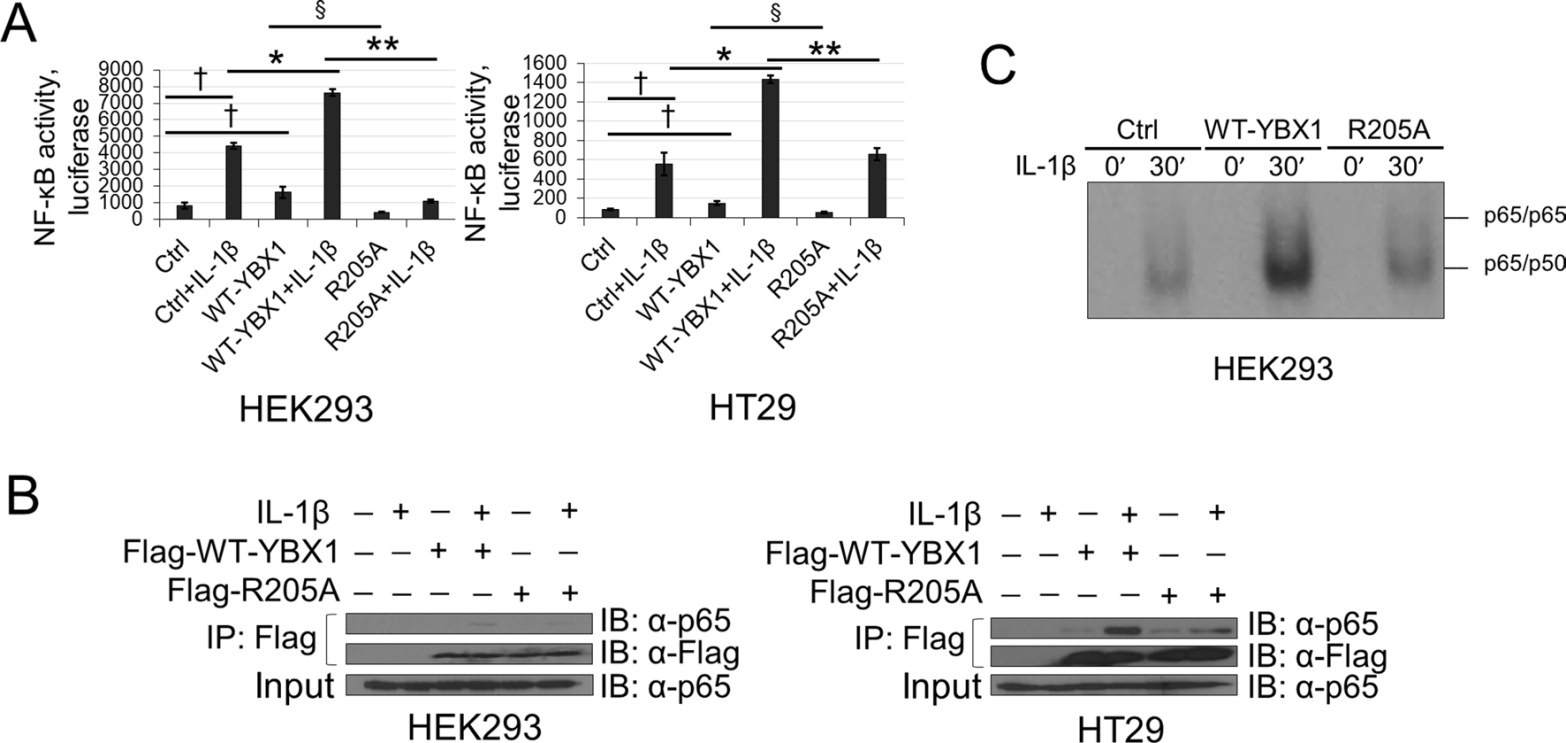
R205A attenuates YBX1-mediated NF-κB activation, p65 DNA binding and complex between p65 and YBX1. (**A**) NF-κB luciferase assay performed in HEK293 (*left*) and HT29 (*right*) cells stably expressing the WT-YBX1 or R205A construct with or without 10 ng/ml IL-1β stimulation. Luciferase readings were normalized to both protein concentration and internal control β-galactosidase. (**B**) Co-immunoprecipitation of YBX1 and endogenous p65. HEK293 and HT29 cells stably overexpressing Flag-WT-YBX1 or Flag-R205A were treated with 10 ng/mL of IL-1β for 1 h or were left untreated. YBX1 primarily complexes with p65 under IL-1β stimulation. Anti-p65 and Anti-Flag IP images were obtained from the same blots that were stripped and reprobed for total Flag but developed at different exposures. Input images were obtained from different blots and developed at different exposures. (**C**) EMSA to determine DNA binding ability of p65 performed with extracts from HEK293 cells overexpressing WT-YBX1 or R205A. Cells were left untreated or stimulated with 10 ng/ml IL-1β for 30mins. The data represent the means ± SD from three independent experiments. ^†^*p* < 0.05 vs. Control (Ctrl) group; *p < 0.05 vs. Ctrl + IL-1β group; ^§^*p* < 0.05 vs. WT-YBX1 group; **p < 0.05 vs. WT-YBX1 + IL-1β group.

Previously, we showed that YBX1 overexpression could significantly augment the activation of NF-κB^10, 11^. We therefore speculated that dimethylated R205 (R205me2) could potentially play an essential role in fine-tuning the activation of NF-κB by YBX1. NF-κB-specific luciferase assays were carried out using the established cell lines described above. As shown in Fig. 4A, upon IL-1β stimulation, NF-κB transactivation was significantly induced in HEK293 and HT29 cells. Overexpression of WT-YBX1 dramatically enhanced this activation potential, whereas cells with overexpression of the R205A mutant demonstrated significantly less activation of NF-κB compared with WT-YBX1, suggesting the importance of R205me2 for the complete activation of NF-κB by YBX1. We then wondered whether the reduced activation of NF-κB observed with the R205A mutant could be indirectly a result of altered protein–protein interactions between p65 and YBX1 and/or impaired downstream DNA binding ability of the prototypical p65/p50 heterodimer of NF-κB. To test the first possibility, we conducted co-immunoprecipitation experiments to compare the extent of the interaction between WT-YBX1 or R205A and p65. Interestingly, as shown in Fig. 4B, IL-1β-induced interaction between YBX1 and p65 was attenuated by the R205A mutant. Next, we investigated the DNA-binding capacity of p65 using a κB-specific electrophoresis mobility gel shift assay (EMSA). Consistent with our previous reports, overexpression of WT-YBX1 further enhanced IL-1β-induced κB binding activity of p65, whereas overexpression of the R205A mutation correlated with an impaired DNA binding ability of p65 compared with WT-YBX1 (Fig. 4C). These data suggest that R205me2 is necessary for the interaction between YBX1 and p65 and may potentially modulate the NF-κB DNA binding ability via this critical protein–protein interaction.

### R205 methylation differentially regulates YBX1-dependent NF-κB target gene expression

**Figure 5:**
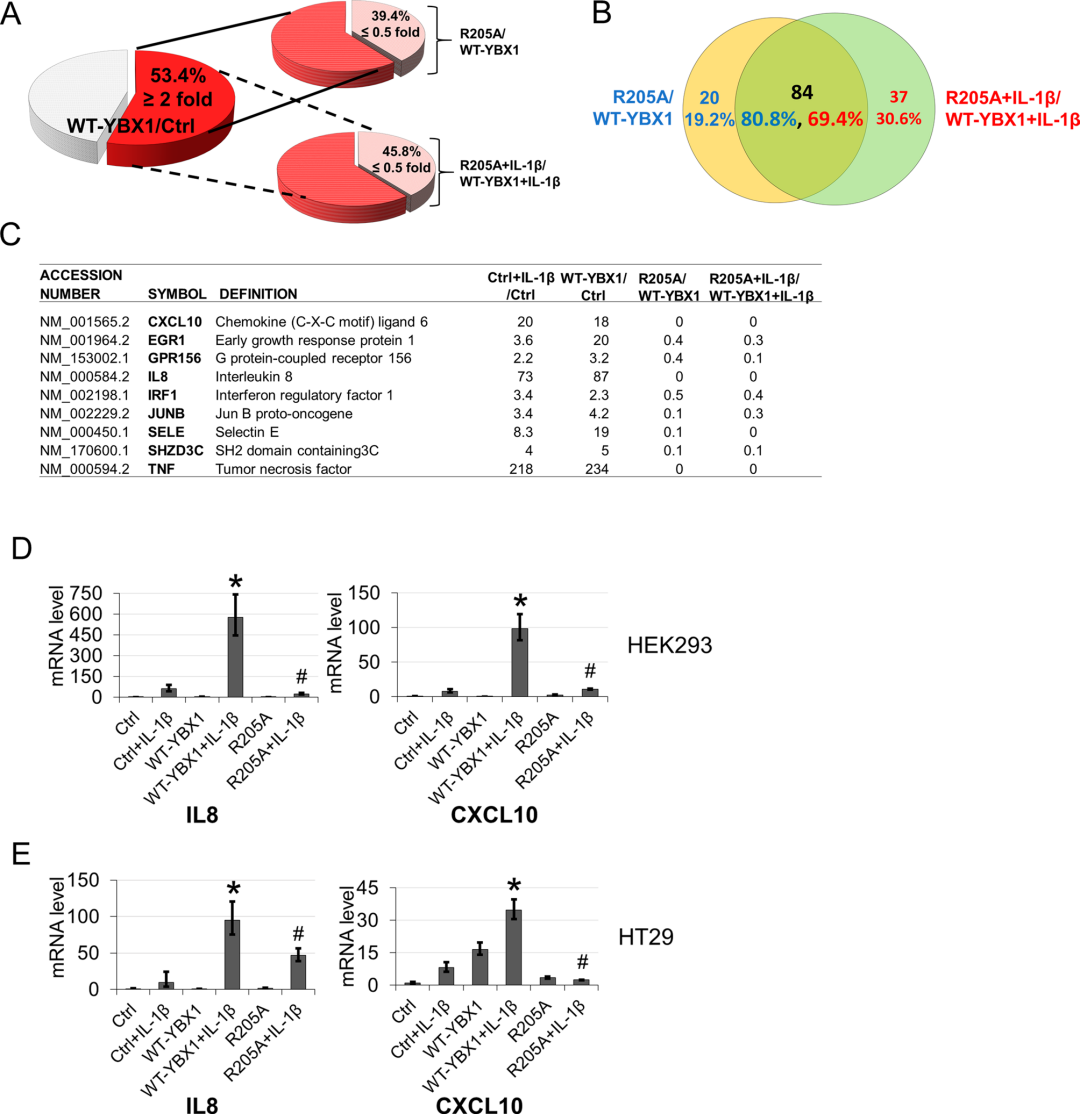
Loss of methylation on R205A results in differential expression of a subset of YBX1-regulated NF-κB target genes. (**A**) Pie chart, *left*, representing comparative analysis of the fraction of genes from vector control (Ctrl) upregulated two-fold or more by IL-1β and within this group, another 53.4% were further augmented by overexpression of WT-YBX1 relative to vector control (WT-YBX1/Ctrl ≥ 2). This group of genes represents the WT-YBX1-dependent NF-κB target genes. Pie chart, *upper right*, shows fraction of WT-YBX1-upregulated genes from the *left pie chart* that were downregulated two-fold or more by R205A overexpression (39.4%) (R205A/WT-YBX1 ≤ 0.5), representing the genes that are regulated by R205A under basal condition. Similarly, lower right pie chart shows fraction of WT-YBX1-upregulated genes from the left pie chart that were downregulated two-fold or more by R205A overexpression under IL-1β stimulated condition (~ 45.8%) (R205A + IL-1β/WT-YBX1 + IL-1β ≤ 0.5). (**B**) Venn diagram, shows 84 genes (black font) commonly regulated by basal (R205A/WT-YBX1) and IL-1β-stimulated (R205A + IL-1β/WT-YBX1 + IL-1β) groups, corresponding to 80.8% of genes under basal condition (blue font) or 69.4% of genes under IL-1β stimulated condition (red font), respectively. Each group also had its own pool of solely regulated genes, such as 20 genes (19.2%) of basal condition (blue font) and 37 genes (30.6%) of IL-1β-stimulated condition, respectively. (**C**) A representative short list of typical IL-1β-inducible NF-κB target genes upregulated by WT-YBX1 but not by R205A under the basal or IL-1β-stimulated conditions. (**C**) Quantitative PCR (qPCR) analysis showing confirmation of two genes from microarray data set. Pro-inflammatory chemokines IL8 and CXCL10 showed reduced gene expression in the mutant R205A as compared to that of WT-YBX1 in HEK293 and (**D**) HT29 cell lines under basal and IL-1β-stimulated conditions. The data represent the means ± SD from at least three independent experiments. *p < 0.05 vs. Ctrl + IL-1β group; ^*#*^*p* < 0.05 vs. WT-YBX1 + IL-1β group.

**Figure 6:**
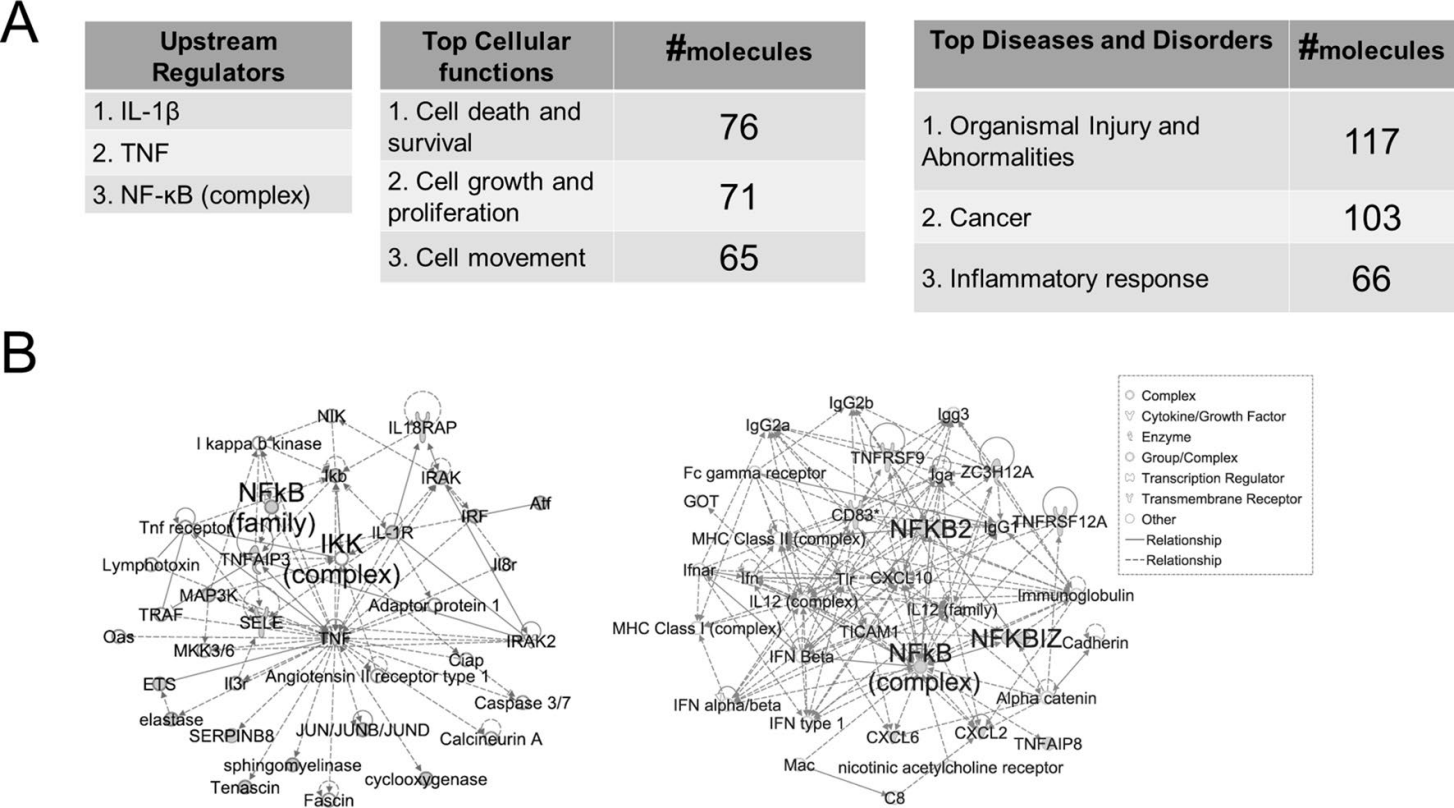
Ingenious pathway analysis (IPA) of genes regulated by R205 methylation and their associated networks and functions. (**A**) Ingenious pathway analysis (IPA), showing list of upstream regulators (IL-1β, TNF and NF-κ) as well as top network cellular functions associated with R205 methylation such as cell death, survival, proliferation and movement (left and middle panel, respectively). The right panel shows that the genes regulated by R205 methylation are associated with networks linked to diseases such as cancer and inflammatory-related disorders. (**B**) Representative networks reveal NF-κB-related proteins as critical nodes in these networks.

Based on our previous findings that overexpression of YBX1 upregulated a discrete subset of NF-κB target genes^11^, we sought to determine if this also occurs in a methylation-dependent manner. To examine this possibility, we conducted an Illumina microarray analysis using HEK293 vector control cells or those overexpressing either WT-YBX1 or R205A in the presence and absence of IL-1β for 4 h. We show that among the total number of NF-κB target genes in HEK293 vector control cells (genes upregulated twofold or more with IL-1β), approximately 53.4% of them could be further upregulated in cells overexpressing WT-YBX1 (Fig. 5A, left pie chart), suggesting these are WT-YBX1-dependent NF-κB genes. Importantly, among these genes, ~ 39.4% of them were downregulated by at least twofold (R205A/WT-YBX1 ≤ 0.5) (Fig. 5A, upper right pie chart), indicating that basal levels of R205 methylation is critical for the expression of ~ 39.4% of WT-YBX1-dependent NF-κB genes. Similarly, we further analyzed the data in IL-1β-treated WT-YBX1 and R205A samples, and observed that approximately 45.8% of WT-YBX1-dependent NF-κB genes were further downregulated twofold or more with the R205A overexpression group (lower right pie chart; R205A + IL-1β/WT-YBX1 + IL-1β ≤ 0.5). This represents an increase (~ 16.2%) of the WT-YBX1-dependent NF-κB genes that are controlled by R205 dimethylation when IL-1β-stimulated. Interestingly but unsurprisingly, cross-comparison of the basal and IL-1β conditions for R205A-downregulated genes revealed that approximately 84 genes were commonly shared between these groups (Fig. 5B), corresponding to the majority, *i.e.* ~ 80.8% of R205me2 regulated genes under basal (unstimulated) condition, and ~ 69.4% of R205me2 regulated genes under IL-1β stimulation, respectively. Importantly, among these genes, as shown in the representative short list in Fig. 5C, we identified several cytokines (Supplementary Fig. 1), chemokines, or adhesion molecules, including tumor necrosis factor (TNF), interleukin 8 (IL8), chemokine (C-X-C motif) ligand 10 (CXCL10), Selectin E (SELE), that are well recognized to be implicated in modulating tumor-host responses and metastasis. Two of these genes, IL8 and CXCL10 were further confirmed by quantitative PCR analysis. These data recapitulated our microarray findings in which WT-YBX1 overexpression significantly augmented mRNA levels of these two genes whereas R205A had the opposite effect in both HEK293 and HT29 cells (Fig. 5D,E). Upon closer analysis of the signature networks associated with this subgroup of genes regulated by R205me2 using an ingenuity pathway analysis (IPA), we observed that the “**top upstream regulators**” were related to IL-1β, TNF and NF-κB (Fig. 6A, *left panel*). Meanwhile, the “**top cellular functions**” were connected to important cellular mechanisms including “cell death and survival”, “cell growth and proliferation” and “cell movement” (Fig. 6A, *middle panel*). Importantly, among the “**top diseases and disorders**”, “cancer” emerged as one of the most enriched networks (Fig. 6A, *right panel*). Interestingly, representative networks in Fig. 6B also uncovered NF-κB as a major hub in these networks. Collectively, these data suggest the significant role of methylation of YBX1 at R205 in mediating activation of a subset of NF-κB target genes with important roles in inflammation, cell survival and cancer.

### R205 methylation is important for the colony formation, cell migration and growth of CRC cells associated with overexpression of YBX1

**Figure 7:**
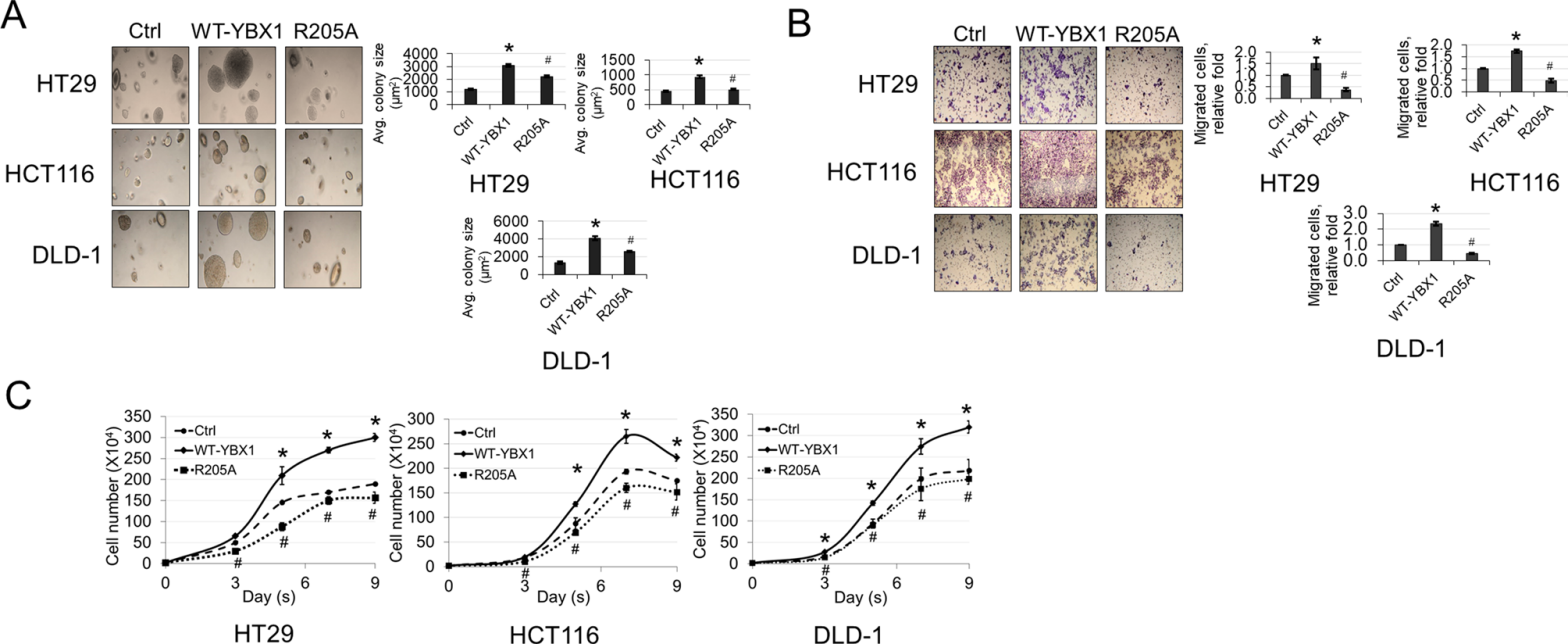
R205A attenuates anchorage-independent growth, migration and proliferation of CRC cells. (**A**) Anchorage-independent growth assays performed in CRC cell lines with vector Ctrl, WT-YBX1 or mutant R205A. Right panel shows quantified data. (**B**) Boyden Chamber cell migration assays performed with same cells as above. Right panel shows quantified data. (**C**) Cell growth assay performed in a panel of CRC cell lines expressing vector Ctrl, WT-YBX1 or mutant R205A. Cell number was assessed at different days over a time course of 9 days. The data represent the means ± SEM or S.D. for at least three independent experiments. *p < 0.05 vs. Ctrl. ^*#*^*p* < 0.05 vs. WT-YBX1.

Based on the network cellular functions enriched in our IPA described above (Fig. 6), we wondered whether the R205A mutant would attenuate any of the malignant-like phenotypes associated with YBX1 overexpression. Hence, we next examined the effects of the R205A mutant on colony formation (anchorage-independent growth), migration and cell growth potential of a panel CRC cell lines (HT29, HCT116, DLD-1). As shown in Fig. 7A, overexpression of WT-YBX1 correlated with the formation of larger colonies compared with the vector control cells, whereas cells with overexpression of R205A showed a significantly reduced colony forming ability compared with WT-YBX1. Furthermore, we observed a similar phenomenon in our Boyden Chamber migration experiments in which overexpression of WT-YBX1 resulted in the migration of a greater number of cells while the R205A mutant significantly attenuated this effect (Fig. 7B). Finally, we also observed in our cell growth assays that the WT-YBX1-oeverexpressing CRC cells had a greater growth potential compared to the vector controls while the R205A cells showed a significant reduction in number compared to WT-YBX1 over a course of 9 days in cell culture (Fig. 7C). Taken together, these data strongly argue for a model in which methylation of YBX1 at R205 modulates its tumor-associated functions in CRC under basal conditions.

### Upregulation of YBX1 and PRMT5 are positively correlated in colorectal adenocarcinoma patient samples

**Figure 8:**
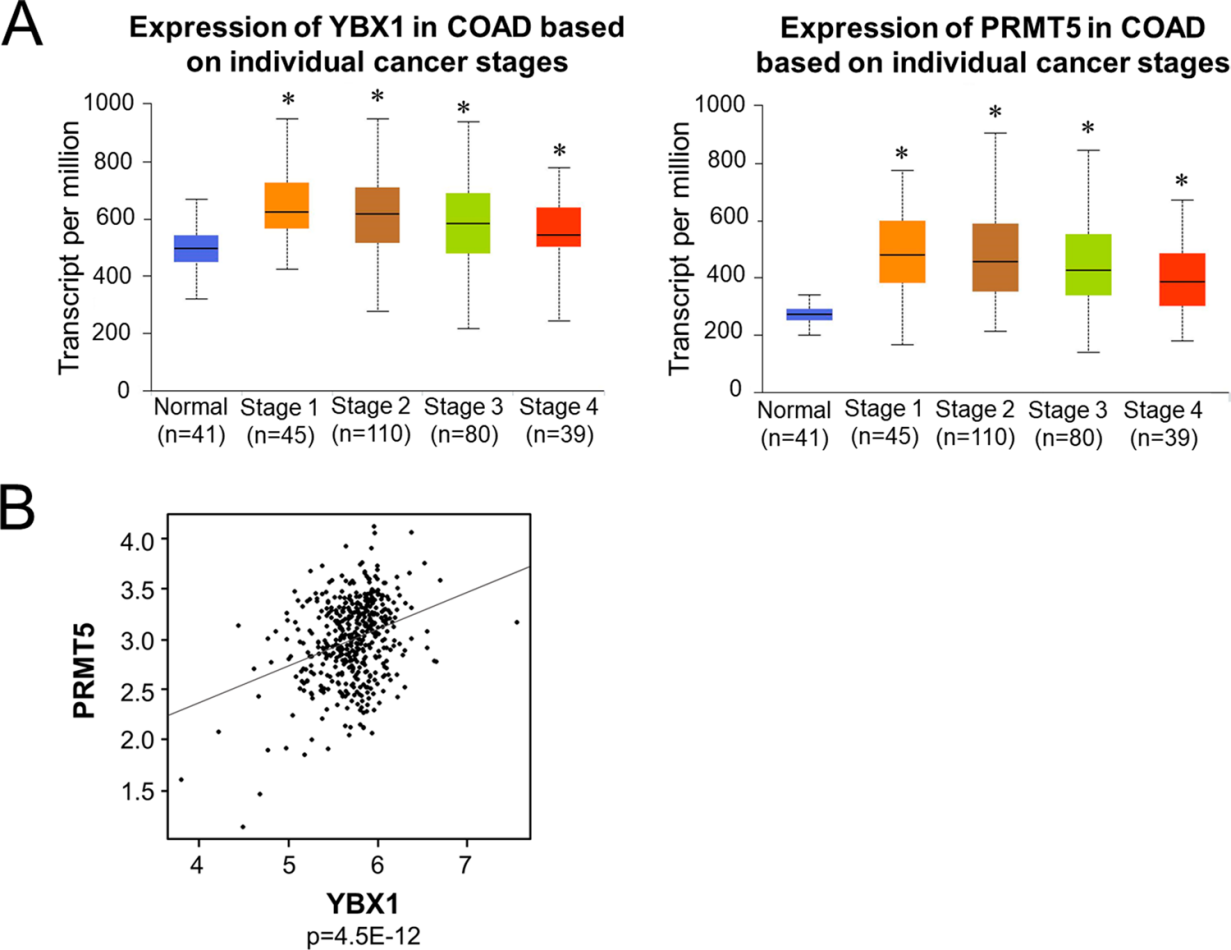
YBX1 and PRMT5 are highly amplified and are significantly correlated in CRC patient samples. (**A**) Box-whisker plots showing transcript levels of YBX1 (*left panel*) and PRMT5 (*right panel*) across colorectal adenocarcinoma (COAD) tumors and normal based on individual cancer stages. Individual cancer stages were based on AJCC (American Joint Committee on Cancer) pathologic tumor stage information and samples were divided into stage I, stage II, stage III and stage IV group. Courtesy of UALCAN web-portal, publicly available at https://ualcan.path.uab.edu. (**B**) Scatter plot showing the expression level of YBX1 (x-axis) and PRMT5 (y-axis). Each dot represents log-transformed fragments per kilobase of transcript per million mapped reads (FPKM) for individual samples. To obtain data, 459 TCGA colorectal adenocarcinoma tumor samples was queried from cBioPortal using R package CGDS-R (https://cran.r-project.org/web/packages/cgdsr). The Pearson correlation was conducted between PRMT5 and YBX1 to detect how much two genes are related. The above analyses were conducted in statistical environment R v3.6 and *p* value less than 0.05 was considered as statistically significant, and Bonferroni correction is used in place of multiple-comparison. R^2^ = 0.04868.

**Figure 9:**
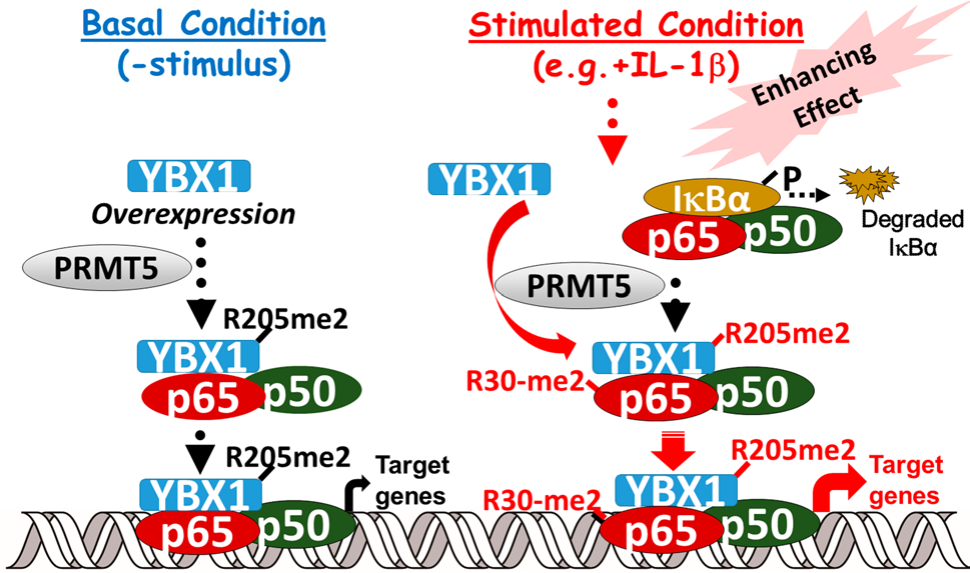
Hypothetical model of differential effects of R205 methylation of YBX1 on NF-κB signaling and promotion of cancer phenotypes. *Left side: *Under basal conditions (without IL-1β stimulation), overexpression of WT-YBX1 activates NF-κB (10, 11) (Figs. 4 and 5). YBX1 is dimethylated on R205, and this is critical for the regulation of a substantial subgroup (~ 39.4%) of WT-*Y*BX1-dependent NF-κB genes. *Right side:* On the other hand, in the presence of stimuli such as IL-1β, the IκB kinase phosphorylates IκBα, causing degradation of IκB. The liberated p65/p50 heterodimer then migrates to the nucleus to bind to cognate κB sequences on the promoters of specific genes, leading to their transcription. While this is occurring, IL-1β can also enhance PRMT5-mediated dimethylation of YBX1 at R205 and simultaneously, dimethylation of p65 at R30 based on previous findings. These two modifications may serve dual purposes. One, the R205me2 modification serves the purpose of enhancing the interaction between p65 and YBX1 and in turn YBX1-mediated activation of p65. Second, methylation of p65 by PRMT5, also serves the function of enhancing the activity and together with YBX1, positively modulates the overall DNA binding capability of p65. These important modifications also positively regulate a subset of NF-κB target genes, i.e. *Y*BX1-R205me2-dependent NF-κB genes. Altogether, the enhanced expression of these genes may act in concert to directly or indirectly promote cancer-related phenotypes which may be alleviated by the disruption of this PRMT5/YBX1/NF-κB signaling axis using PRMT5 inhibitors.

CRC is in part characterized by uncontrolled cell growth as a consequence of the coordinated upregulation of certain oncogenic drivers, many of which have served as useful biomarkers^27^. In this respect, we sought to further analyze whether the upregulated of YBX1 and PRMT5 are positively correlated in CRC patient samples. We found that not only is YBX1 upregulated or mutated across a broad spectrum of cancers (Supplementary Fig. 2), but its transcript levels (Fig. 8, *left panel*) are also significantly higher across colorectal adenocarcinoma (COAD) tumors compared to normal tissue counterparts. Using the same The Cancer Genome Atlas (TCGA) data, we observed a similar phenomenon for PRMT5 (Fig. 8, *right panel*). Interestingly, we also observed that the expression level of YBX1 (x-axis) and PRMT5 (y-axis) were significantly positively correlated in COAD patients, suggesting that this YBX1-PRMT5 correlation could serve as the basis for future patient stratification and identification of patients who are likely to benefit from inhibition of YBX1-PRMT5 cooperativity. The underlying mechanism of this cooperativity is summarized in our hypothetical model in Fig. 9.

## Discussion

The findings outlined in this work constitute the first report of methylation of YBX1. We have demonstrated that PRMT5-mediated methylation of YBX1 at R205 plays an essential role in the regulation of a subgroup of YBX1-dependent NF-κB target genes under both basal (unstimulated) (~ 39.4% of those genes, Fig. 5A), and IL-1β stimulated conditions (~ 45.8% of those genes, Fig. 5A). The R205 methylation of YBX1 is important for its interaction with p65, revealing a novel cooperativity between YBX1 and PRMT5 that dynamically modulates NF-κB activity and target gene expression to render a tumor-promoting effect in CRC cells. Moreover, our discovery that the expression levels of these two tumor-associated proteins are positively correlated in CRC patients’ samples is novel and noteworthy. Both YBX1 and PRMT5 have emerged as important therapeutic targets for several cancers including CRC and are thought to act upstream of cellular signaling pathways involved in tumorigenesis and cancer progression^21, 28^. PRMT5 in particular has especially garnered increasing attention lately. Its methylation of arginine residues on both histone and nonhistone proteins has been intrinsically linked to many cellular processes to which YBX1 has also been linked such as differentiation, signal transduction, splicing, and cell cycle progression^21, 28^. Importantly, similar to YBX1, PRMT5 is frequently overexpressed in CRC and other human cancers, and its expression is positively correlated with disease progression and poor patient outcomes^25, 29^. Hence, this newly discovered PRMT5/YBX1 axis may potentially serve as an important biomarker and basis for patient stratification. Future studies toward elucidating the potential therapeutic benefit of inhibiting both YBX1 and PRMT5 will be of great importance in this respect.

Besides the above-discussed aspects, the interaction between PRMT5, YBX1 and NF-κB is likely more complex and exquisite than what our data have captured so far. For instance, we previously reported that PRMT5 interacts with and can also symmetrically dimethylate the R30 residue of p65, an event that is also triggered by IL-1β^17, 30^. In the current work, we have modeled YBX1 as one target protein of PRMT5 in a similar context, however, this model may not present a full picture of what PRMT5 will do in the presence of multiple substrates simultaneously. Hence, based on our previous reports, it is likely that both YBX1 and p65 are coordinately methylated by PRMT5 in our system. One could also speculate that IL-1β may act as a tool to enhance the assembly of a larger protein complex in which PRMT5 is brought into close proximity with YBX1, in turn allowing for its R205 methylation and association with p65. Meanwhile, PRMT5 could also methylate p65 at R30, which together with the YBX1-R205me2-mediated activation of p65, serves the dual purpose of amplifying the NF-κB response. These are important questions that require further experimental investigation. Importantly, addressing these points will also allow us to better understand one outstanding question in the field. What are the factors that govern PRMT5 substrate specificity? Given the complexity of PRMT5 interactions and its multiple nonhistone substrates, fully characterizing these factors and understanding their link to observed cancer-associated effects will be of paramount importance as we seek to understand the functions of PRMT5 and the consequences of its inhibition.

In addition to deciphering the afore-mentioned points, we also acknowledge that the exact mechanism by which YBX1, and particularly methylated YBX1 may enhance NF-κB DNA-binding ability is still unknown. Raj et al.^31^ have shown preliminary evidence that YBX1 may potentially modulate p65 transactivation by augmenting its affinity for consensus κB sequences. In the present study, we have now confirmed by EMSA that YBX1-mediated enhancement of this p65 DNA binding occurs in a YBX1 methylation-dependent manner. However, how this occurs remains unclear. As suggested by Lorton et al.^32^, the overall main effect of arginine methylation is alteration of protein–protein, protein-DNA, and protein-RNA interactions of the methylated protein. This is predicted to be the consequence of new *van der Waals* contacts that stabilize intra-protein interactions which may indirectly increase the affinity of the methylated protein to nucleic acids^17^. With regards to the current study, these findings are intriguing on many levels. First, YBX1 binds both DNA and RNA and we have now shown that its interaction with p65 depends on methylation at R205, implying that methylation of YBX1 at R205 may potentially affect some of the cellular and biochemical properties of YBX1 previously assigned to residues in the vicinity of the methylated residue. Second, this coincides with data from other groups showing that indeed, the C-terminal region of YBX1, where the R205me2 residue is located, is also primarily responsible for the sequence-nonspecific binding of YBX1 to DNA and RNA and mediation of protein–protein interactions^33^. It would therefore be of immense interest to understand how methylation of YBX1 may play a role in not only facilitating the YBX1/p65 interaction but whether it may also concomitantly enhance the efficiency of p65 binding at its consensus DNA sequences based on the indiscriminate but cooperative binding of YBX1 to DNA structures near the κB binding sites.

Furthermore, based on previous reports of PRMT5′s and YBX1′s roles in splicing, our discovery may also have novel implications for these proteins’ collaborative regulation of the splicing machinery and/or splicing products^4, 22^. More in-depth explorations to address these notions by determining where and how PRMT5, YBX1 and p65 interact (in the cytoplasm, nucleus, and spliceosome) are warranted. We anticipate that techniques such as cell fractionation-immunoprecipitation, chromatin immunoprecipitation (ChIP) and in silico modeling coupled with iCLIP-seq analyses will be valuable approaches in this regard.

Finally, since YBX1 has also been shown to synergistically interact with other transcription factors (TFs), including AP-1^34^, p53^2^ and Smad3^35^ it would be worth investigating if R205me2 serves as a general PTM mechanism for regulating the interaction between YBX1 and these TFs or if this might be a p65-specific phenomenon. This knowledge will also unquestionably deepen our understanding of how a single PTM event on YBX1 or how possible crosstalk with other TF-PTM circuits can direct the transcription of a specific set of target genes. Fully understanding the mechanisms underlying such complex interactions may unravel novel therapeutic avenues for CRC and other tumor types.

## Methods

### Cell lines and transfections

The HEK293C6 (293) cell line was previously described^36^. CRC cell lines HT29, DLD1 and HCT116 were purchased from ATCC (Manassas, VA) and were cultured in RPMI (Roswell Park Memorial Institute) 1640 medium supplemented with 100 units/ml penicillin, 100 g/ml streptomycin, and 5% fetal bovine serum (FBS). The Myc- or Flag-R205A mutant of YBX1 was generated using the QuikChange II XL Site-Directed Mutagenesis Kit from Agilent Technologies. Primers were designed using the Agilent Technologies QuikChange Primer Design online software. Constructs were transfected into HEK293C6 and CRC cell lines as essentially described by Lu et al.^36^ using Lipofectamine and PLUS reagents (Life Technologies/Invitrogen).

### Digestion and liquid chromatography-tandem mass spectrometric analysis

A Coomassie-stained SDS-PAGE gel band containing the YBX1 protein was subjected to in-gel tryptic digestion as previously described^36^. Following destaining procedures (50% acetonitrile in 100 mm ammonium bicarbonate and 100% acetonitrile), cysteine residues were reduced using 20 mm DTT at room temperature for 60 min. Alkylation of the sample was then performed with 55 mm iodoacetamide, 30 min in the dark. Gel pieces were then washed with 100 mm ammonium bicarbonate, dehydrated in acetonitrile and dried in a SpeedVac centrifuge. Trypsin (Promega, WI) was used to rehydrate and digest the sample, overnight at 37 °C. Extracted peptides were reconstituted in 0.1% formic acid and prepared for mass spectrometry analysis. Proteolytic digests were analyzed using an LTQ Orbitrap XL linear ion-trap mass spectrometer (Thermo Fisher Scientific) coupled with an Ultimate 3000 HPLC system (Dionex). The data were analyzed by Mascot software (Matrix Science) against customized YBX1 protein database with the setting of 10 ppm for precursor ions and 0.8 Da for product ions. The tandem mass spectra of candidate-modified peptides were further interpreted manually.

### Western blotting and antibodies

Cells were cultured to about 90–95% confluence before treatment with IL-1β (10 ng/ml). Whole cell samples were collected and lysed using Radio Immunoprecipitation Assay buffer (RIPA buffer: 150 mM NaCl, 0.1% Triton X-100, 0.5% sodium deoxycholate, 0.1% sodium dodecyl sulfate (SDS), 50 mM Tris–HCl pH 8.0 and protease inhibitors)^36^. Whole cell lysates were then separated by SDS/PAGE gels, and further assessed by Western blotting. Different antibodies were used to detect the target proteins of interest, obtained from the following commercial sources: anti-YBX1 (Abcam, ab12148) anti-PRMT5 (Abcam, ab109451), anti-Flag (Sigma-Aldrich, F1804), anti-Myc (Cell Signaling, 71D10) and anti-p65 (Santa Cruz Biotechnology, sc-109).

### Luciferase assays

NF-κB luciferase assays were conducted as previously described^36^. Briefly, lentivirus made from the κB-luciferase construct p5XIP10 κB, envelope plasmid pMD2.G and packaging plasmid pCMV8.91 was used to infect vector control, Flag-WT-YBX1 or Flag-R205A stable cell lines after which luciferase activity was quantified 48 h later using the Luciferase Assay System with Reporter Lysis Buffer kit (Promega). The κB-luciferase plasmid p5XIP10 κB contains five tandem copies of the NF-κB DNA binding site derived from the IP10 gene (an established target gene of NF-κB) upstream of a luciferase reporter gene. Luciferase activity was measured using a Synergy H1 Multi-Mode Reader (BioTek Instruments Inc.).

### Co-immunoprecipitations

Cells were cultured to 95% confluency then lysed in co-immunoprecipitation buffer (1% Triton X-100 (v/v), 50 mM Tris–HCl (pH 7.4), 150 mM NaCl, 1 mM EDTA, 1 mM sodium orthovanadate, 20 μM aprotinin, 1 mM phenylmethanesulfonyl fluoride, and 1 mM pepstatin A). The Flag or Myc-YBX1 proteins were immunoprecipitated with anti-Flag-M2 beads (Sigma-Aldrich, A2220) or anti-Myc-beads (Sigma, E6654), using immunoprecipitation methods previously described. Briefly, cell lysates with equivalent amounts of protein were incubated with appropriate beads at 4 °C overnight. Beads were then washed and tagged proteins were eluted and separated by SDS/PAGE.

### Cell growth and anchorage-independent growth assays

For cell growth assays, vector ctrl or CRC cells overexpressing Flag-WT-YBX1 or R205A constructs were seeded in triplicate at 2 × 10^4^ cells/well in a 6-well plate. Cells were counted at days 3, 5, 7 and 9 post-seeding using a cell counting chamber. For anchorage-independent growth assays, type VII agarose (Sigma) was used to prepare 2.4% and 1.2% bottom and top agar layers, respectively. 2 × 10^5^ cells were resuspended in the top layer and plated onto the bottom layer. Cells were then cultured for 12–14 days at 37 °C and 5% CO2. Images of colonies were captured using a Canon EOS Rebel T3i Digital SLR camera and colony size and number were quantified using ImageJ (https://imagej.nih.gov/ij/).

### Migration assay

Migration assays were conducted using Boyden chambers. Briefly, a Boyden chamber consists of 8 μm pore size cell culture inserts in a 24 well plate. Each insert was coated with gelatin on the side facing the lower chamber. 2 × 10^5^ cells were seeded in the top of the insert (upper chamber) in serum-free media while serum-rich media (10% serum) was supplied in the well below as a chemoattractant. After 48 h, migrated cells were fixed with 4% formaldehyde and stained with crystal violet. Stained cells were visualized with a light microscope and quantified. Images were captured using a Canon EOS Rebel T3i Digital SLR camera.

### Immunofluorescence assay

Immunofluorescence assays were carried out as previously described^11^. 1 × 10^5^ HEK293 cells overexpressing Flag-WT-YBX1 were seeded in a 24-well plate unto a 0.1% sterile gelatin pre-coated coverslip/well and left overnight. Cells were fixed with 4% formaldehyde solution for 30 min followed by blocking buffer for 10 min at R.T. Coverslips were further probed with anti-Flag antibody for the detection of Flag-tagged WT-YBX1 and anti-PRMT5 antibody for the detection of endogenous PRMT5 followed by Alexa Fluor 488 (green) goat anti-mouse IgG and Alexa Fluor 594 goat anti-rabbit IgG. Coverslips were sealed using mounting media containing HOECHST to stain the nucleus. All slides were visualized under a Leica DMI6000B series fluorescent microscope at 63 × magnification.

### Illumina microarrays and quantitative PCR (qPCR)

Microarray and qPCR experiments were carried out as essentially described by Wei et al.^17^. Briefly, cells were cultured to ~ 90% confluence and total RNA was isolated using Trizol reagent. Total isolated RNA was used to prepare cDNA using the SuperScript III First-Strand Synthesis PCR System (Invitrogen). cDNA was labeled with biotin-UTP using the Illumina Total Prep RNA amplification kit (Ambion/Applied Biosystems, Foster City, CA), hybridized to Illumina Human Ref-v3 v1 Expression Bead Chips and then scanned in a Bead Array reader using standard Illumina protocols (Illumina, San Diego, CA). Illumina’s Bead Studio software was used for data analysis. qPCR was carried out using FastStart Universal SYBR Green Master ROX (Roche). Primers were designed by the Primer Express 3.0 software. Primer information is listed below: CXCL10-Forward: 5′- TGAAAAAGAAGGGTGAGAAGAGATG-3′; CXCL10-Reverse: 5′-CCTTTCCTTGCTAACTGCTTTCAG-3′; IL8-Forward: 5′-TCCTGATTTCTGCAGCTCTGT-3′; IL8-Reverse: 5′-AAATTTGGGGTGGAAAGGTT-3′.

### Cytokine array

The human cytokine ELISA array was purchased from Signosis, Inc. (Santa Clara, CA USA). Experiments were carried out according to the manufacturer’s protocol. Briefly, cells from HT29 control, WT-YBX1- and R205A—overexpressing stable cell lines were seeded and cultured for 72 h. Conditioned media from these cells was collected by centrifugation and added to specific cytokine capture antibody precoated wells for 2 h at R.T. Wells were then washed the luminescent signals were detected using an HRP luminescent substrate and Synergy H1 Multi-Mode Reader (BioTek Instruments Inc.). The luminescent signal emitted corresponded to the relative level of expression for each specific cytokine detected.

### Ingenuity pathway analysis (IPA)

Groups of genes regulated by R205A were analyzed by the IPA software^11^. The setting and filter were as follows: reference set: Ingenuity Knowledge Base (Genes _ Endogenous Chemicals); Relationship to include: Direct and Indirect; Includes Endogenous Chemicals; Filter Summary: Consider only molecules where species _ Human OR Rat OR Mouse. The *p* values for the enrichment test were calculated using Fisher’s exact test, right-tailed. Log10 (*p*) was visualized to the left of the *p* value. *p* < 0.05 was considered to be statistically significant.

### Correlation analyses

The RNA-seq for 459 TCGA colorectal adenocarcinoma tumor samples was queried from cBioPortal using R package CGDS-R (https://cran.r-project.org/web/packages/cgdsr/). The gene expression data was then log transformed and used for correlation analysis between YBX1 and PRMT5. The Pearson correlation was conducted between genes to detect how much they are related. The above analyses were conducted in statistical environment R v3.6 and *p* value less than 0.05 was considered as statistically significant, and Bonferroni correction is used in place of multiple-comparison.

### Statistical analysis

Statistical analyses were performed using Prism 6 software (GraphPad, San Diego, CA). Data represent the mean ± S.D. or SEM as indicated. A two-tailed Student’s *t-*test was used when comparing two means between groups as specified. All statistics were carried out for triplicate experiments and a *p* < 0.05 was considered statistically significant.

## Supplementary information

Supplementary Information 1.

Supplementary Information 2.
