# Supplementary material for: PRMT5-mediated methylation of YBX1 regulates NF-κB activity in colorectal cancer
Source: Sci Rep. 2020 Sep 28;10:15934. doi: 10.1038/s41598-020-72942-3 (PMC7522246; doi:10.1038/s41598-020-72942-3)

PRMT5-mediated methylation of YBX1 regulates NF-κB activity in colorectal cancer

Antja-Voy Hartley[1], Benlian Wang[2], Rasika Mundade[1], Guanglong Jiang[4], Mengyao Sun[1], Han Wei[1], Steven Sun[1], Yunlong Liu[4] & Tao Lu*[1,3,4].

[1] Department of Pharmacology & Toxicology, Indiana University School of Medicine, Indianapolis, IN; [2]Case Western Reserve University, Cleveland, OH; [3]Department of Biochemistry & Molecular Biology, Indiana University School of Medicine, Indianapolis, IN; [4]Department of Medical & Molecular Genetics, Indiana University School of Medicine, Indianapolis, IN

*Corresponding author:

Tao Lu: Department of Pharmacology and Toxicology, Indiana University School of Medicine, 635 Barnhill Drive, Indianapolis, IN 46202, USA

Tel: 317-278-0520

Fax: 317-274-7714

Email: lut@iu.edu

Running title: PRMT5 methylates YBX1 at R205

NON-PUBLSIHED SUPPLEMENTAL INFORMATION

<u>FIGURE 2A –</u> WB: Anti-YBX1 and Anti-beta-actin

HEK293: Anti-YBX1 (left)

HT29: Anti-YBX1 (middle)

HT29 and HEK293, respectively: Anti-beta-actin (right)

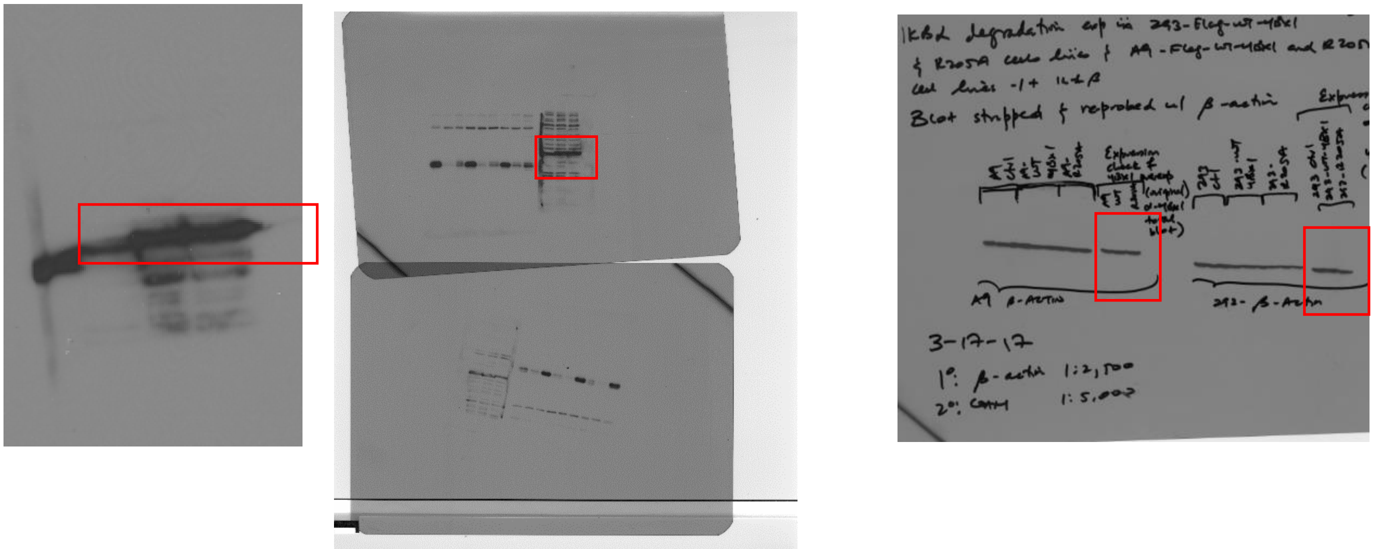

<u>FIGURE 2B HEK293-</u> IP: Anti-SDMA and IP: Anti-Myc (-/+ IL-1) were cropped from the same blot (10 sec exposure) stripped and reprobed with Anti-Myc. Input: Anti-Myc and Input: Anti-YBX1 were developed from different blots. Blots were developed along with multiple blots on the same film. Relevant parts of each blot are shown.

IP: Anti-SDMA (-/+ IL-1) (top left)

IP: Anti-Myc (-/+ IL-1) (top right)

Input: Anti-Myc (-/+ IL-1) (bottom left)

Input: Anti-YBX1 (-/+ IL-1) (bottom right)

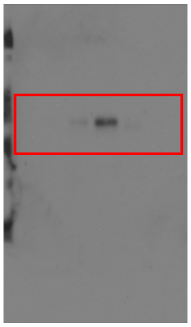

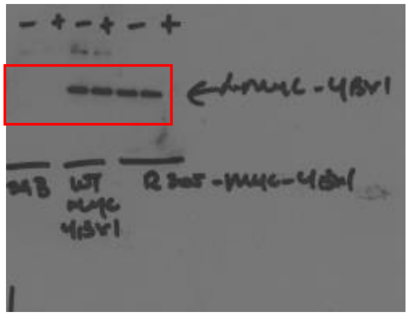

Short exposure

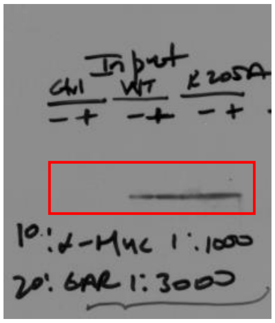

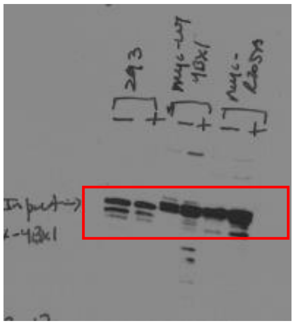

Long exposure

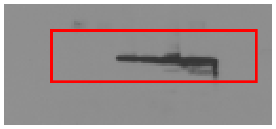

<u>FIGURE 2B HT29 -</u> IP: Anti-SDMA and IP: Anti-Myc (-/+ IL-1) were cropped from the same blot (10 sec exposure) stripped and reprobed with Anti-Myc. Input: Anti-Myc and Input: Anti-YBX1 were developed from different blots. Blots were developed along with multiple blots on the same film. Relevant parts of each blot are shown.

IP: Anti-SDMA (-/+ IL-1) (top left)

IP: Anti-Myc (-/+ IL-1) (top right)

Input: Anti-Myc (-/+ IL-1) (bottom left)

Input: Anti-YBX1 (-/+ IL-1) (bottom right)

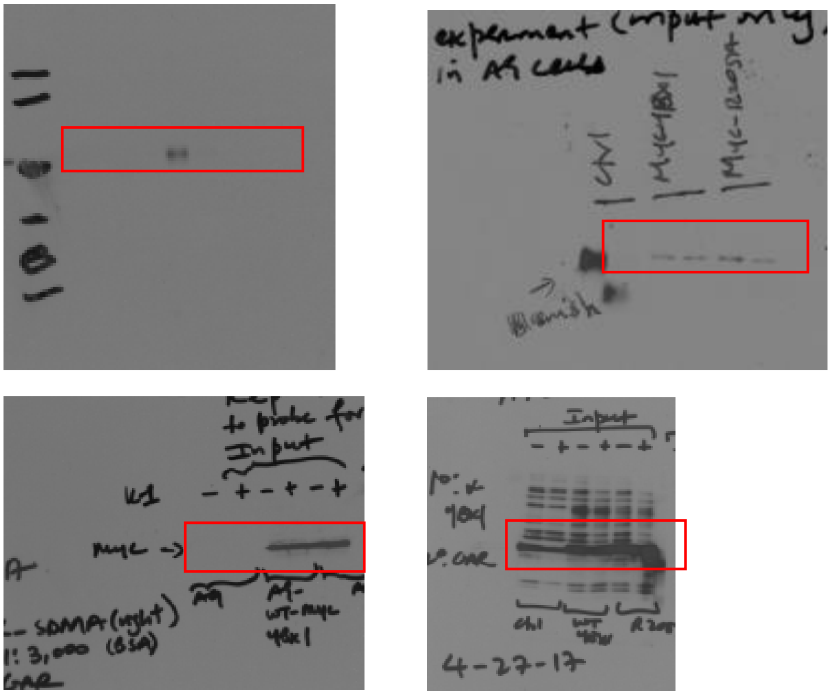

<u>FIGURE 3A - </u>IP: Anti-Myc and Input: Anti-Myc (-/+ IL-1) were cropped from the same blot (2 sec exposure) while the IP: Anti-Flag image was developed from the same blot that was stripped and re-probed with anti-Flag antibody (10sec exposure).

IP: Anti-Myc and Input: Anti-Myc (-/+ IL-1) (left)

IP: Anti-Flag (-/+ IL-1) (right)

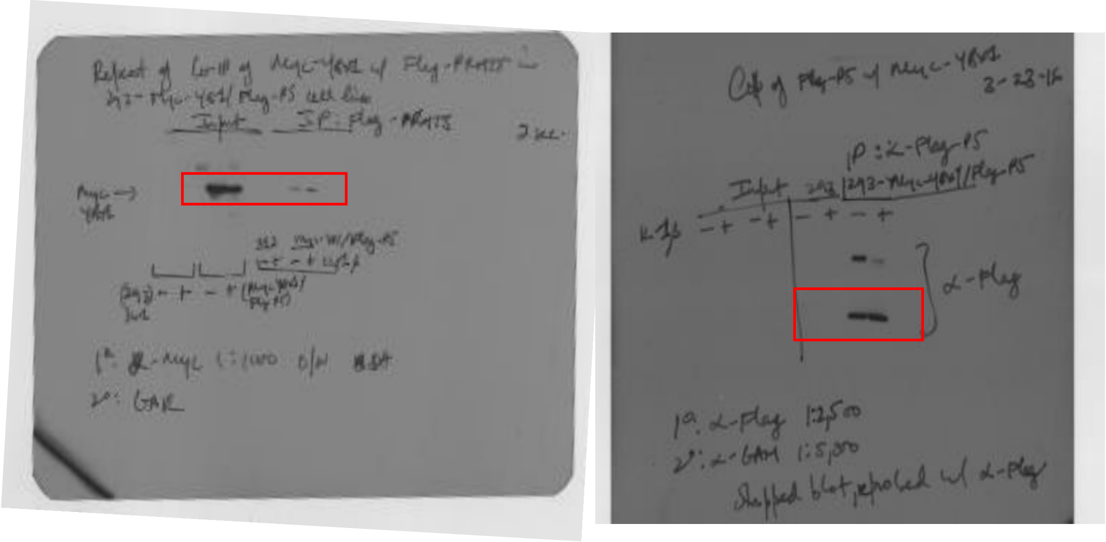

FIGURE 3B – Images were developed on blots with multiple other blots. Relevant part(s) of the blots shown. IP: Anti-Myc and Input: Anti-Myc (-/+ IL-1) were cropped from two different blots (1min exposure) while the IP: Anti-Flag image was developed from the same blot as IP: Anti-Myc that was stripped and re-probed with Anti-Flag antibody (1 min exposure). Note: samples were run alongside Myc-R205A samples which are shown in blots below but not in the main text Figures.

IP: Anti-Myc (-/+ IL-1) (left)

IP: Anti-Flag (-/+ IL-1) (middle)

Input: Anti-Myc (-/+ IL-1) (right)

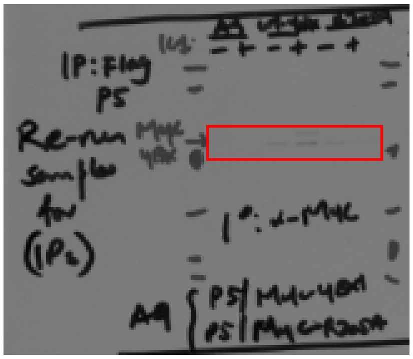

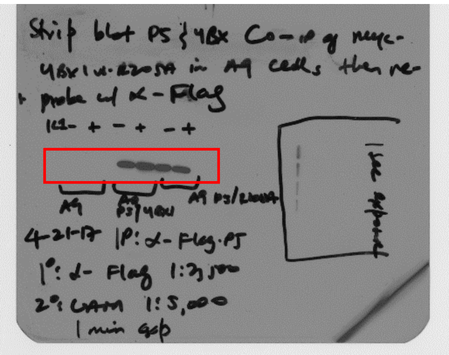

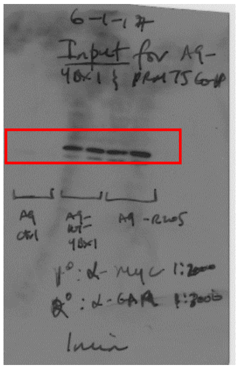

FIGURE 3C

(Left) Images were developed same blot as other samples. Relevant part(s) of the blots shown. WB: anti-PRMT5. The (right) WB for anti-actin was obtained from same blot stripped and reprobed.

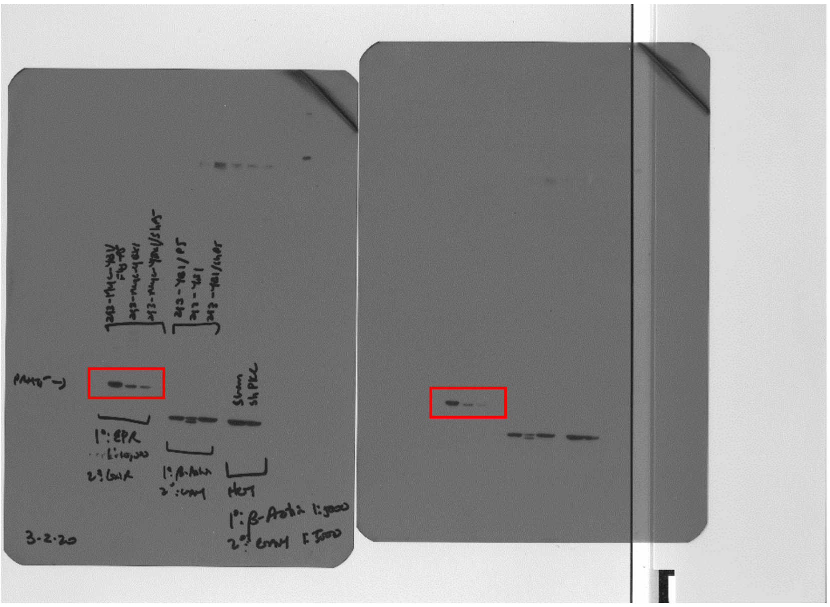

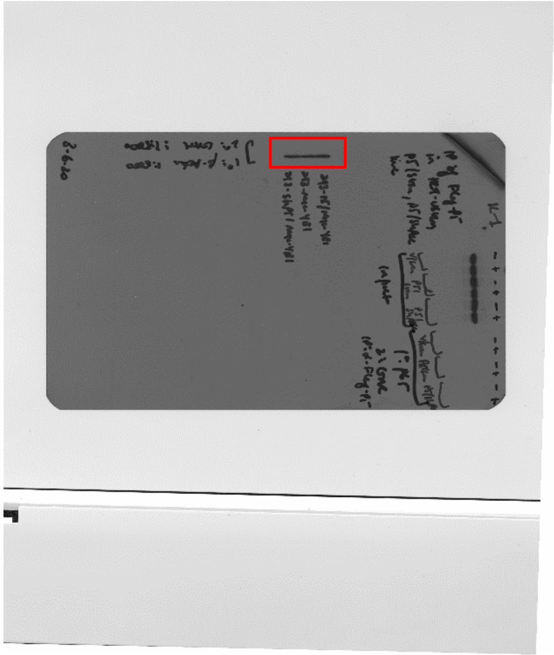

FIGURE 3D

Images were developed on blots with multiple other blots. Relevant part(s) of the blots shown. IP: Anti-SDMA and Input: Anti-Myc (-/+ IL-1) were cropped from two different blots (1min) while the IP: Anti-YBX1 image was developed from the same blot as Input: Anti-Myc that was stripped and re-probed with Anti-YBX1 antibody (1 min exposure). The IP: Anti-Myc image was obtained from the IP: Anti-SDMA blot that was stripped and re-probed with Anti-Myc antibody.

IP: Anti-SDMA (-/+ IL-1) (top left)

IP: Anti-Myc (-/+ IL-1) (top right, long and short exposure)

Input: Anti-Myc (-/+ IL-1) (bottom left)

Input: Anti-YBX1 (-/+ IL-1) (bottom right)

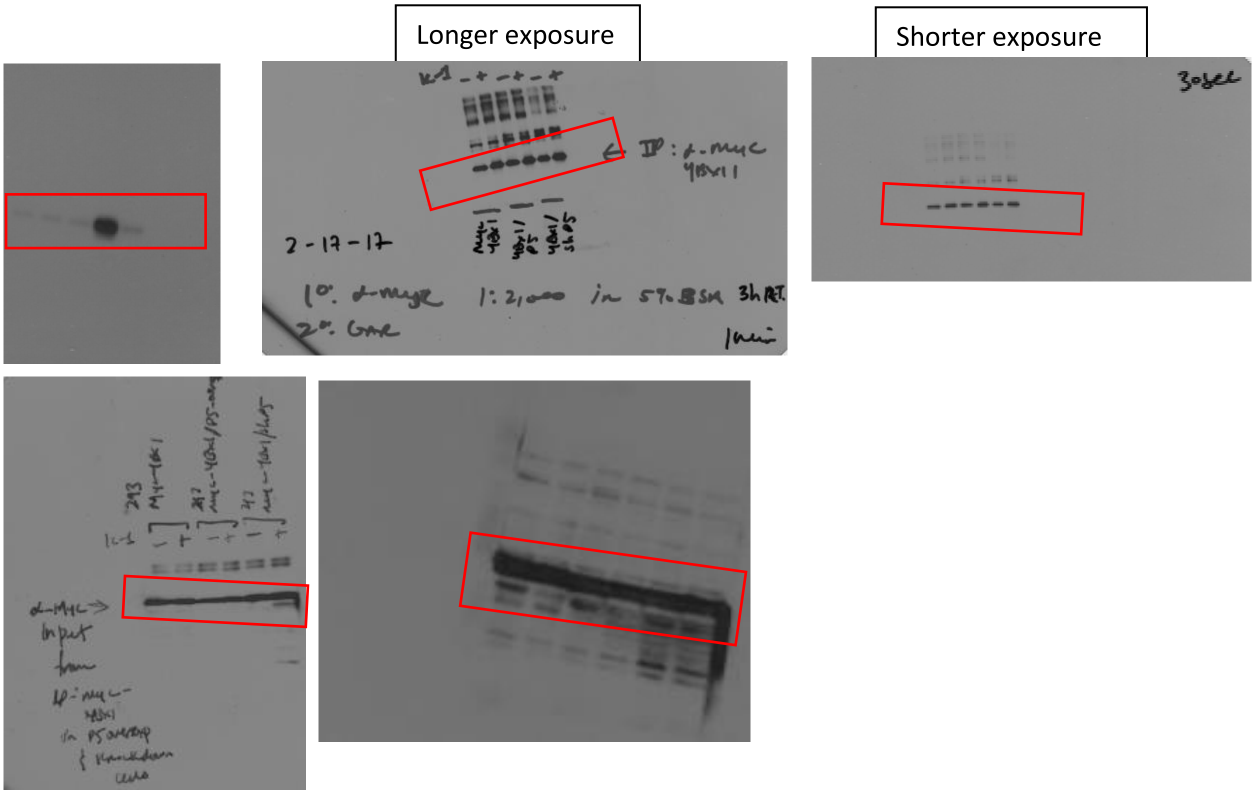

FIGURE 4B (HEK293) - IP: Anti-p65 and IP: Anti-Flag (-/+ IL-1) were cropped from the same blot (30 sec exposure) that was stripped and re-probed with Anti-Flag. Input: Anti-p65 image was developed from a different blot (10sec exposure). Blots were developed with other blots on the same film and the relevant part of each blot is shown.

IP: Anti-p65 (-/+ IL-1) (left)

IP: Anti-Flag (-/+ IL-1) (middle)

Input: Anti-p65 (-/+ IL-1) (right)

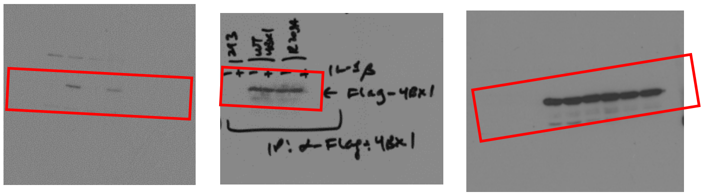

<u>FIGURE 4B (HT29) -</u> IP: Anti-p65 and IP: Anti-Flag (-/+ IL-1) were cropped from the same blot (30 sec exposure) that was stripped and re-probed with Anti-Flag. Input: Anti-p65 image was developed from a different blot (10sec exposure). Blots were developed with other blots on the same film and the relevant part of each blot is shown.

IP: Anti-p65 (-/+ IL-1) (left)

IP: Anti-Flag (-/+ IL-1) (middle)

Input: Anti-p65 (right)

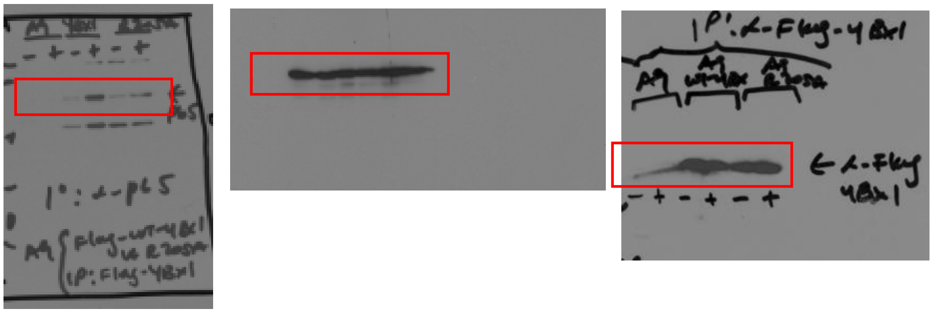

Supplement: Supplementary file 1 — Supplementary Information 1. [file 41598_2020_72942_MOESM1_ESM.pdf]
