# Supplementary material for: PRMT5-mediated methylation of YBX1 regulates NF-κB activity in colorectal cancer
Source: Sci Rep. 2020 Sep 28;10:15934. doi: 10.1038/s41598-020-72942-3 (PMC7522246; doi:10.1038/s41598-020-72942-3)
Supplement: Supplementary file 2 — Supplementary Information 2. [file 41598_2020_72942_MOESM2_ESM.pdf]

# PRMT5-mediated methylation of YBX1 regulates NF-κB activity in colorectal cancer

Antja-Voy Hartley[1], Benlian Wang[2], Rasika Mundade[1], Guanglong Jiang[4], Mengyao Sun[1], Han Wei[1], Steven Sun[1], Yunlong Liu[4] & Tao Lu*[1,3,4].

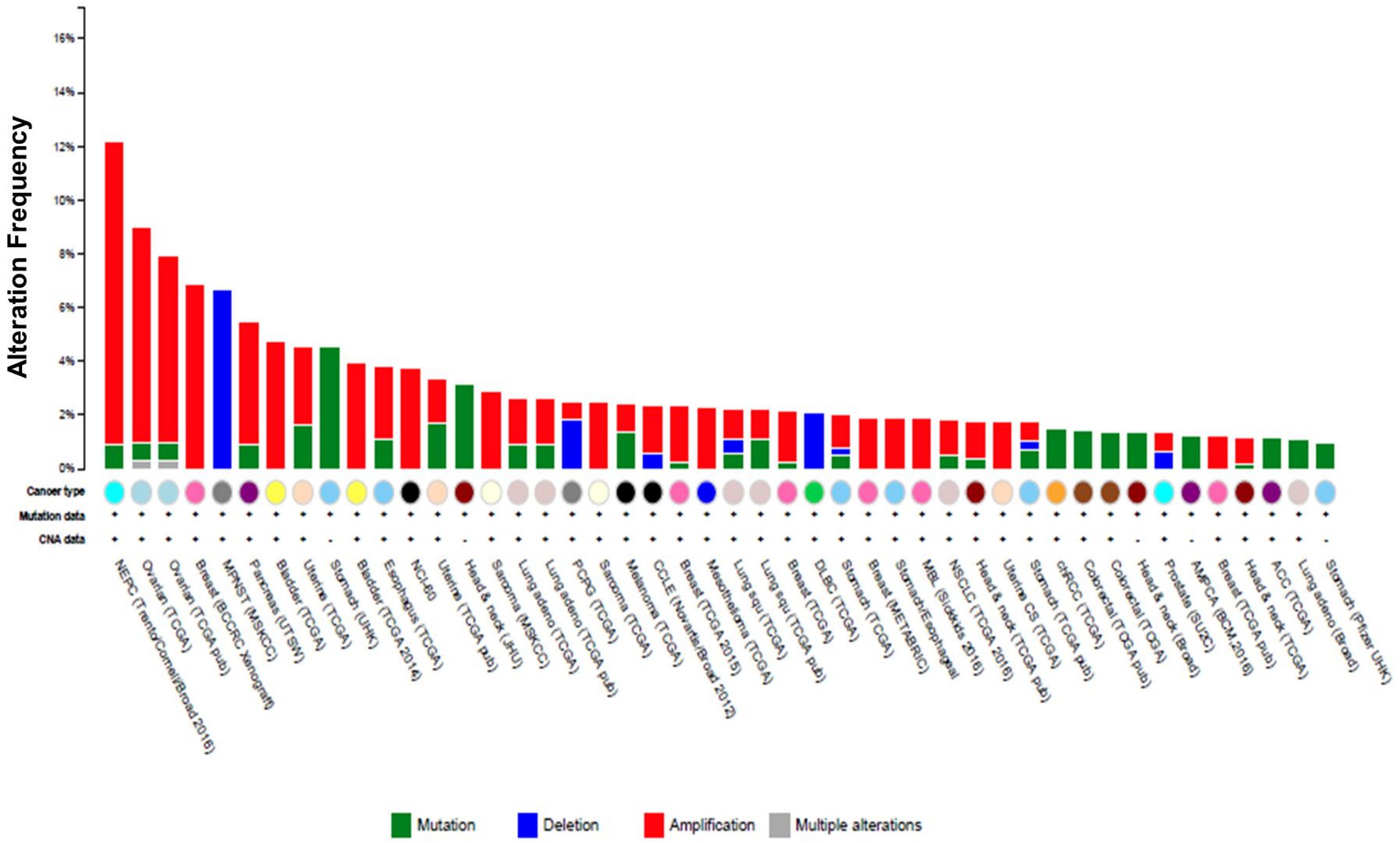
Alteration Frequency
16%
14%
12%
10%
8%
6%
4%
2%
0%
Cancer type
Mutation data
CNA data
NEPC (Trento/Cornell/Broad 2016)
Ovarian (TCGA)
Ovarian (TCGA pub)
Breast (BCCRC Xenograft)
MPNST (MSKCC)
Pancreas (UTSW)
Bladder (TCGA)
Uterine (TCGA)
Stomach (UHK)
Bladder (TCGA 2014)
Esophagus (TCGA)
NCI-60
Uterine (TCGA pub)
Head & neck (JHU)
Sarcoma (MSKCC)
Lung adeno (TCGA)
Lung adeno (TCGA pub)
PCPG (TCGA)
Sarcoma (TCGA)
Melanoma (TCGA)
CCLE (Novartis/Broad 2012)
Breast (TCGA 2015)
Mesothelioma (TCGA)
Lung squ (TCGA)
Lung squ (TCGA pub)
Breast (TCGA)
DLBC (TCGA)
Stomach (TCGA)
Breast (METABRIC)
Stomach/Esophageal
MBL (Sickkids 2016)
NSCLC (TCGA 2016)
Head & neck (TCGA pub)
Uterine CS (TCGA)
Stomach (TCGA pub)
chRCC (TCGA)
Colorectal (TCGA pub)
Colorectal (TCGA)
Head & neck (Broad)
Prostate (SU2C)
AMPCA (BCM 2016)
Breast (TCGA pub)
Head & neck (TCGA)
ACC (TCGA)
Lung adeno (Broad)
Stomach (Pfizer UHK)
Mutation
Deletion
Amplification
Multiple alterations

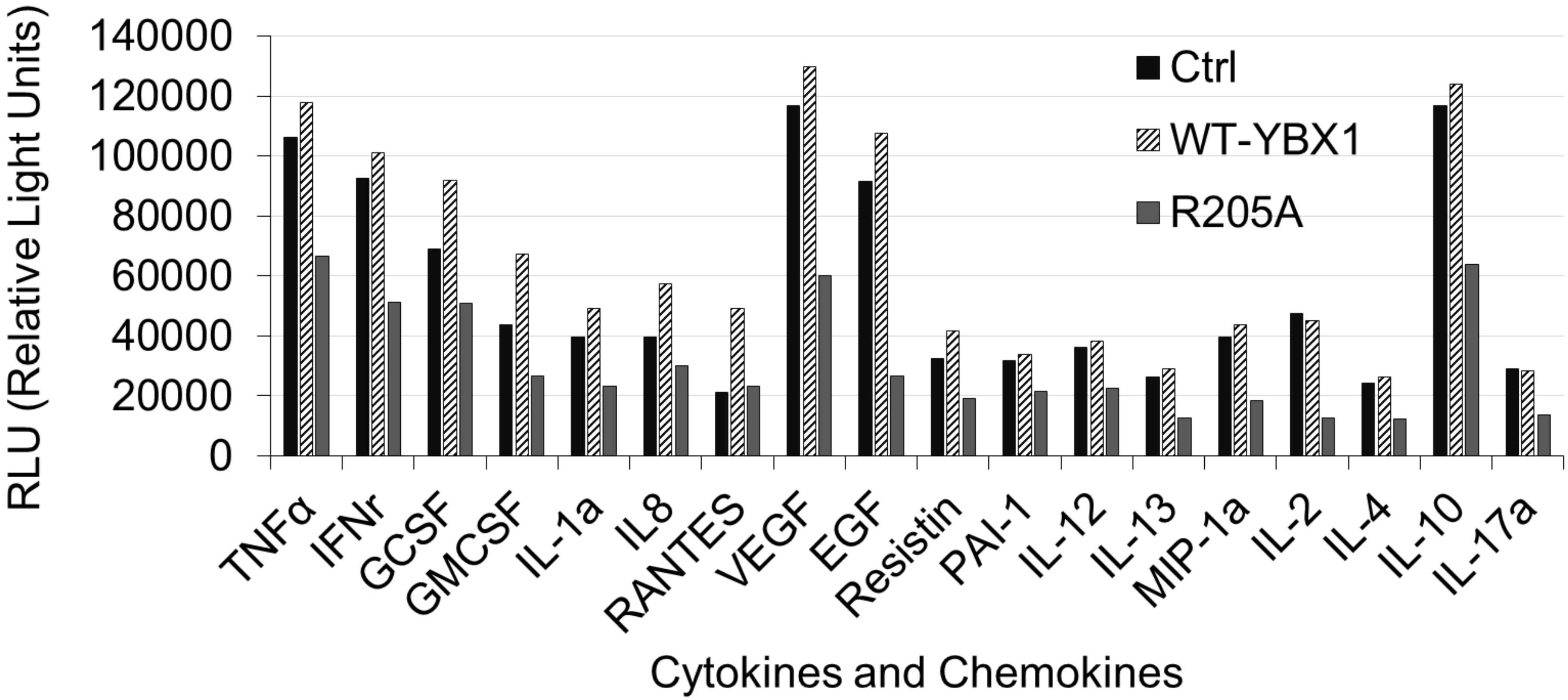

RLU (Relative Light Units)
140000
120000
100000
80000
60000
40000
20000
0
Ctrl
WT-YBX1
R205A
TNFα
IFNr
GCSF
GMCSF
IL-1a
IL8
RANTES
VEGF
EGF
Resistin
PAI-1
IL-12
IL-13
MIP-1a
IL-2
IL-4
IL-10
IL-17a
Cytokines and Chemokines
